# Natural history and genotype‐phenotype correlation of pantothenate kinase‐associated neurodegeneration

**DOI:** 10.1111/cns.13294

**Published:** 2020-02-11

**Authors:** Xuting Chang, Jie Zhang, Yuwu Jiang, Jingmin Wang, Ye Wu

**Affiliations:** ^1^ Department of Pediatrics Peking University First Hospital Beijing China

**Keywords:** genotypic spectrum, natural history, *PANK2*, Pantothenate kinase‐associated neurodegeneration disease

## Abstract

**Aims:**

To investigate the natural history and genotype‐phenotype correlation of pantothenate kinase‐associated neurodegeneration.

**Methods:**

We collected data of patients with PKAN by searching from available publications in English and Chinese. Patients diagnosed in our center (Peking University First Hospital) were also included. The difference in natural history and genotype between early‐onset (<10 year of age at onset) and late‐onset patients (≥10 year of age at onset) with PKAN was compared.

**Results:**

A total of 248 patients were included. The median age at onset was 3.0 years in the early‐onset group and 18.0 years in the late‐onset group. Dystonia in lower limbs was the most common initial symptom in both groups. In the early‐onset group, the median interval between the disease onset and occurrence of oromandibular dystonia, generalized dystonia, loss of independent ambulance was 6.0 years, 5.0 years, and 5.0 years. The corresponding values in late‐onset group were 1.0 year, 4.0 years, and 6.0 years. About 20.0% died at median age of 12.5 years and 9.5 years after the onset in early‐onset group. About 2.0% of the late‐onset patients died during the follow‐up. A total of 176 mutations were identified. Patients carrying two null alleles in *PANK2* showed significantly earlier age of disease onset and progressed more rapidly to loss of independent ambulance.

**Conclusions:**

This study provided a comprehensive review on the natural history and genotype of 248 patients with PKAN. The results will serve as a historical control data for future clinical trial on PKAN.

## 1 INTRODUCTION

**Figure 1:**
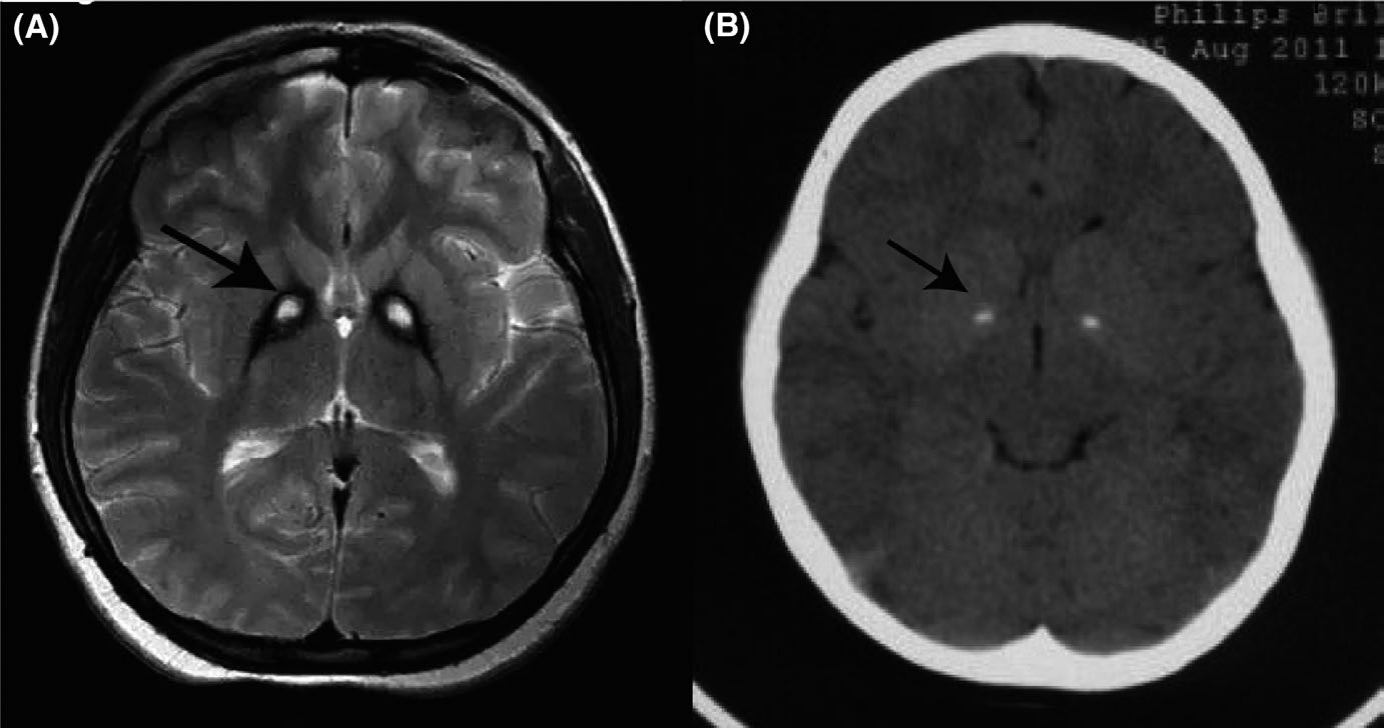
Neuroimaging of patients with PKAN. A, T2‐weighted MRI of the brain showed a specific pattern of hyperintensity (indicated by the arrow) within the hypointense medial globus pallidus (“eye‐of‐the‐tiger” sign). B, Brain CT showed calcification in medial globus pallidus, which was indicated by the arrow

Pantothenate kinase‐associated neurodegeneration (PKAN, OMIM#234200, formerly known as Hallervorden‐Spatz syndrome), also called neurodegeneration with brain iron accumulation 1 (NBIA1), is the main form of NBIA.^1^ The two subtypes of PKAN include classic and atypical. Classic patients are usually characterized by dystonia before 10 years of age and loss of ambulation 10‐15 years after onset. Atypical patients are characterized by dystonia and dysarthria after 10 years of age, and they cannot walk independently until 15‐40 years after onset.^2^ However, there are patients with early onset but slow progression or late onset with rapid progression. The typical manifestation of neuroimaging is the “eye‐of‐the‐tiger” sign (Figure 1). In 2001, biallelic recessive mutations in *PANK2* were revealed in PKAN.^3^ The mutations result in a decreased activity of pantothenic acid kinase 2, which is a key regulatory enzyme in coenzyme A production by pantothenic acid, possibly leading to the reduction of coenzyme A^3^ and accumulation of its substrates, cysteine, which may chelate iron accumulation.^4^ The reported prevalence was 1‐3/1 000 000,^4^ but the accurate prevalence of PKAN is unclear yet.

Understanding of the natural history of PKAN is essential for decision‐making on disease management by physicians and setups of future clinical trials. However, there are few large sample studies. We summarized the natural history and genotypic spectrum of 248 patients with PKAN by searching and analyzing the data from available publications. Patients diagnosed in our center (Pediatric Department, Peking University First Hospital) were also included and pooled for the analysis. We compared the differences in natural history and genotype between early‐onset and late‐onset patients with PKAN.

## 2 METHODS

### 2.1 Inclusion of patients

#### 2.1.1 Literature searching strategy

Case series studies or case reports on PKAN containing information on the clinical and genotypic spectra of patients were searched from the following databases: PubMed, Embase, Cochrane Library, Web of Science, China National Knowledge Infrastructure (CNKI), and WAN FANG DATA (the latter two are Chinese public searching databases). The following search terms were used: pantothenate kinase‐associated neurodegeneration, PKAN, PANK2, Hallervorden‐Spatz syndrome, NBIA, or neurodegeneration with brain iron accumulation. The publication was in English or Chinese. The searching deadline was July 5, 2019.

#### 2.1.2 Inclusion criteria of patients from our center

Patients who met all the following criteria were enrolled: (a) dystonia; (b) “eye‐of‐the‐tiger” sign on brain MRI; and (c) biallelic pathogenic or likely pathogenic variants in *PANK2*. The study was approved by the Medical Ethics Committee of Peking University First Hospital. Informed consent was obtained from the parents of the children.

### 2.2 Data collection

The following information of the patients was collected: basic information (gender, age, and country), symptoms at onset and during the progression, examination (fundus, cranial MRI, and cranial CT), and mutations in *PANK2* in each individual. Patients were divided into early‐onset (<10 year of age at onset) and late‐onset groups (≥10 years of age at onset).

### 2.3 Statistical analysis

The enumeration data were expressed as frequency and percentage. The measurement data were expressed as median (min‐max). Chi‐square test was performed to compare the difference of genotype between the early‐ and late‐onset patients. Mann‐Whitney U test was conducted to evaluate the relationship between the genotype and age of onset, and to compare the differences in time of occurrence of the major symptoms between the early‐ and late‐onset patients. *P* < .05 was considered statistically significant. Survival analysis was used to compare the progression of motor disability between the early‐ and late‐onset patients and the progression among the patients with 0, 1, and 2 null alleles as well. Variables with a significance level < .1 in the survival analysis were then tested in multivariate logistic regression analysis. Because detailed clinical data were not described in some of the studies, we used “n” to represent the number of available patients for the analysis. We summarized the available data when we analyzed the proportion, age of symptoms, and the interval between disease onset and symptoms. Missing data were removed when regression analysis was performed. When we analyzed the distribution of null allele in early‐onset patients and late‐onset patients, and the correlation between the age of onset and genotype, and multivariate logistic regression was performed, we excluded cases with only one mutation detected.

## 3 RESULTS

### 3.1 General information of the patients

A total of 248 patients (132 males and 116 females) were included. Of these patients, 242 were from 66 reported studies (Supporting Information), and 6 were from our center. Of the 248 patients, 128 were early‐onset patients, and 120 were late‐onset patients.

Among the early‐onset patients, the median age at onset was 3.0 (0.3‐9.0, n = 127) years. The age at the last follow‐up described in the literatures was 12.0 (1.8‐60.0, n = 101) years, with 8.3 (0.0‐54.0) years after the onset. While among the late‐onset patients, the median age at onset was 18.0 (10.0‐52.0, n = 120) years. The age at the last follow‐up was 32.0 (12.0‐65.0, n = 102) years, with 10.0 (0.0‐40.0) years after the disease onset.

### 3.2 Phenotypic features of the early‐onset and late‐onset patients

#### 3.2.1 Onset of the disease

As for the initial symptom, dystonia in lower limbs was the most common in both early‐ and late‐onset patients, followed by dystonia in upper limbs. Some patients initially presented with vision loss, generalized dystonia, involuntary movement, or cognitive impairment.

Developmental delay (DD) was more common in early‐onset patients before disease onset. DD was seen in 36.4% (24/66) of the early‐onset patients. Of the 25 patients with onset before 2 years of age, DD was the initial symptom in 5 patients, whereas majority of late‐onset patients (95.5%, 42/44) showed normal developmental milestones before disease onset.

#### 3.2.2 Progression of dystonia

**Figure 2:**
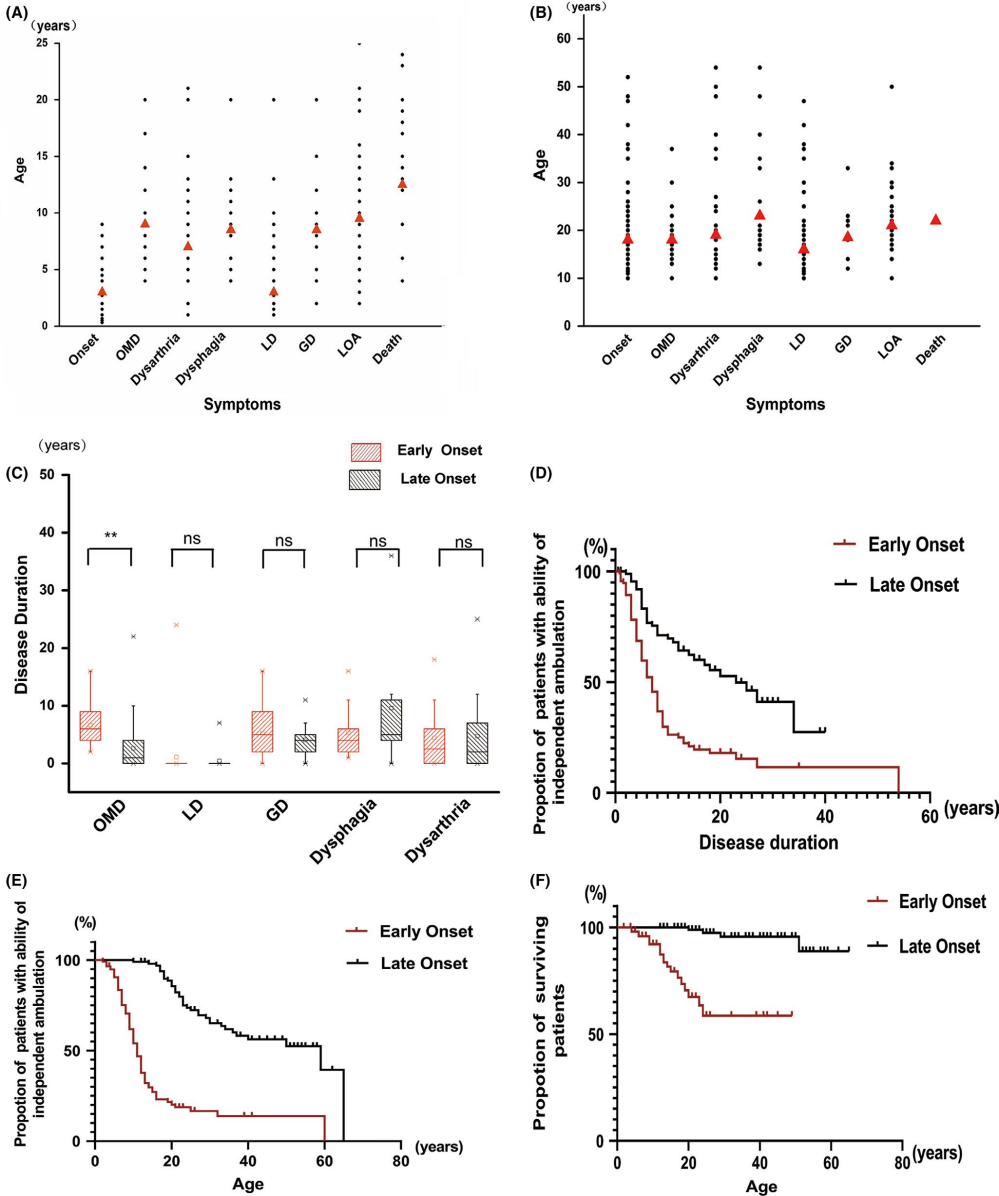
Comparison of disease progression between the early‐onset and late‐onset patients. (A,B) represented the scatter plots of the symptoms in the disease course of the early‐onset (A) and late‐onset patients (B), respectively. The abscissa represented the symptoms, and the ordinate denoted the age at which each symptom was shown. The dot corresponded to each individual, and the red triangle indicated the median. OMD, oromandibular dystonia; LD, limb dystonia; GD, general dystonia; and LOA, loss of independent ambulation (C). The comparison of the time of occurrence for each symptom after disease onset between early‐onset and late‐onset patients. **denotes *P* < .05, and “ns” indicates no statistical difference. D, Survival curve of ability of independent ambulation from disease onset in early‐onset and late‐onset patients. E, Survival curve of ability of independent ambulation with age in early‐onset and late‐onset patients. F, Survival curve of survival patients with age in early‐onset and late‐onset patients

The occurrence of dystonia of limbs, oromandibular dystonia, generalized dystonia, and loss of function of independent walking was compared between the early‐ and late‐onset patients, respectively (Table1, Figure2). Dystonia of limbs occurred in almost all patients, regardless of early‐onset (97/100, 97.0%) or late‐onset patients (95/109, 87.2%), and usually as the initial symptom at onset. Oromandibular dystonia was developed in 80% (68/85) of early‐onset and 56.3% (54/96) of late‐onset patients. Symptom of oromandibular dystonia occurred significantly earlier after disease onset in the late‐onset group (1.0 year, 0.0‐22.0) than in the early‐onset group (6.0 years, 2.0‐13.0) (*P* < .001). About 76.7% (66/86) of early‐onset and 47.5% (47/99) of late‐onset patients progressed to general dystonia before the last follow‐up. The interval between disease onset and general dystonia was comparable in early‐ (5.0 years, 0.0‐16.0) and late‐onset groups (4.0 years, 1.0‐11.0). The proportion of patients who lost their ability to walk independently was significantly higher in the early‐onset group (86/120, 71.7%) than in the late‐onset group (38/105, 36.2%). Moreover, early‐onset patients progressed more rapidly to loss of independent ambulance than late‐onset patients after disease onset (5.0 years, 0.0‐54.0, vs 6.0, 0.0‐20.0 years, *P* < .001). About 74.7% (71/95) of early‐onset patients and 31.3% (21/67) of late‐onset patients could not walk independently within 10 years after disease onset.

**Table 1:** Phenotypic features of PKAN in early‐ and late‐onset patients

Symptoms Median (Min‐Max) (years)	Early‐onset N = 128	Late‐onset N = 120	P value
Age of disease onset	3.0(0.3‐9.0)	18.0(10.0‐52.0)	/
Course of disease at the last follow‐up	8.3 (0.0‐54.0)	10.0 (0.0‐40.0)	/
Dystonia of limbs			
Prevalence %(n/n)	97.0% (97/100)	87.2% (95/109)	.005
Age of onset	3.0 (1.0‐28.0)	16.0 (10.0‐47.0)	/
Time from disease onset	0.0 (0.0‐24.0)	0.0 (0.0‐24.0)	1.000
Oromandibular dystonia			
Prevalence %(n/n)	80.0% (68/85)	56.3% (54/96)	.001
Age of onset	9.0 (4.0‐20.0)	18.0 (10.0‐37.0)	/
Time from disease onset	6.0 (2.0‐13.0)	1.0 (0.0‐22.0)	<.001
Generalized dystonia			
Prevalence %(n/n)	76.7% (66/86)	47.5% (47/99)	<.001
Age of onset	9.0 (2.0‐20.0)	18.5 (12.0‐33.0)	/
Time from disease onset	5.0 (0.0‐16.0)	4.0 (1.0‐11.0)	.646
Dysphagia			
Prevalence %(n/n)	67.3% (35/52)	31.9% (22/69)	<.001
Age of onset	8.5 (4.0‐20.0)	23.0 (13.0‐54.0)	/
Time from disease onset	5.0(1.0‐16.0)	5.0(0.0‐36.0)	.243
Dysarthria			
Prevalence %(n/n)	86.4% (89/103)	80.0% (80/100)	.297
Age of onset	7.5 (1.0‐20.0)	19.0 (10.0‐54.0)	/
Time from disease onset	3.0 (0.0‐18.0)	2.0 (0.0‐25.0)	.964
Loss of independent ambulation			
Prevalence %(n/n)	71.7% (86/120)	36.2% (38/105)	<.001
Age of onset	9.5 (2.0‐60.0)	21.0 (10.0‐50.0)	/
Time from disease onset	5.0 (0.0‐54.0)	6.0 (0.0‐20.0)	<.001
Psychological and behavior problems			
Prevalence %(n/n)	44.9% (48/107)	38.8% (38/98)	.412
Cognitive decline			
Prevalence %(n/n)	54.0% (34/63)	33.8% (24/71)	.025
Pyramidal sign			
Prevalence %(n/n)	62.7% (74/118)	39.7% (31/78)	<.001
Pigmentary retinopathy			
Prevalence %(n/n)	58.5% （48/82）	15.4%（8/52）	<.001
Death			
Prevalence %(n/n)	20.0% (20/100)	2.0% (2/101)	<.001
Age of death	12.5 (4.0‐24.0)	20.0, 24.0	/
Time from disease onset	9.5 (2.0‐23.0)	4.0, 14.0	/

Note

“/” means no statistical analysis was performed. Data were showed Prevalence %(n/n) or median (range). Statistical analysis included Mann‐Whitney U test, survival analysis and chi‐square test, as appropriate.

#### 3.2.3 Bulbar function

Dysphagia and dysarthria were assessed to represent the bulbar function (Table1, Figure2). Dysarthria was more common than dysphagia in both groups. Dysarthria occurred in 86.4% (89/103) of early‐onset and 80.0% (80/100) of late‐onset patients, whereas dysphagia was shown in 67.3% (35/52) and 31.9% (22/69), respectively. The interval between the disease onset and the occurrence of bulbar symptoms was comparable in the two groups. Dysarthria was shown 3.0 (0.0‐18.0) years after the disease onset in early‐onset patients and 2.0 (0.0‐25.0) years in late‐onset group (*P* > .05). Dysphagia occurred after 5.0 (1.0‐16.0) years in early‐onset and 5.0 (0.0‐36.0) years in late‐onset patients (*P* > .05).

#### 3.2.4 Psychological and behavior disturbance

Compulsive behavior, emotional liability, anxiety, depression, or attention deficiency was reported in both early‐onset and late‐onset groups, with slightly more common in early‐onset patients. About 44.9% (48/107) of early‐onset and 38.8% (38/98) of late‐onset patients were reported to present with psychological and behavior problems (*P* = .412).

#### 3.2.5 Cognitive decline

The assessment of cognitive function may be interfered by the limited mobility in PKAN. After the disease onset, cognitive decline was reported in 54.0% (34/63) of the early‐onset patients and 33.8% (24/71) of the late‐onset patients (*P* = .025).

#### 3.2.6 Other signs or symptoms

Pyramidal signs were more prevalent in early‐onset patients (74/118, 62.7%) than late‐onset patients (31/78, 39.7%) (*P* < .001). Pigmentary retinopathy was reported in 58.5% (48/82) of the early‐onset patients and 15.4% (8/52) of the late‐onset patients (*P* < .001).

#### 3.2.7 Survival of the patients

Among the early‐onset patients, twenty patients (20/100, 20.0%) died during the follow‐up at median age of 12.5 (4.0‐24.0) years and 9.5 (2.0‐23.0) years after the onset. While among the late‐onset patients, two (2/101, 2.0%) died at 20 and 24 years of age, with 4.0 and 14.0 years after the onset, respectively.

#### 3.2.8 Brain MRI and CT

“Eye‐of‐tiger sign” in brain MRI was reported in 76.4% (97/127) and 95.5% (105/110) of early‐onset and late‐onset patients, respectively. Few studies reported calcification in globus pallidus. Seven out of eleven patients (63.6%) showed calcification in brain CT. All patients with calcification harbored biallelic mutations.

### 3.3 Genotype‐phenotype correlation

**Figure 3:**
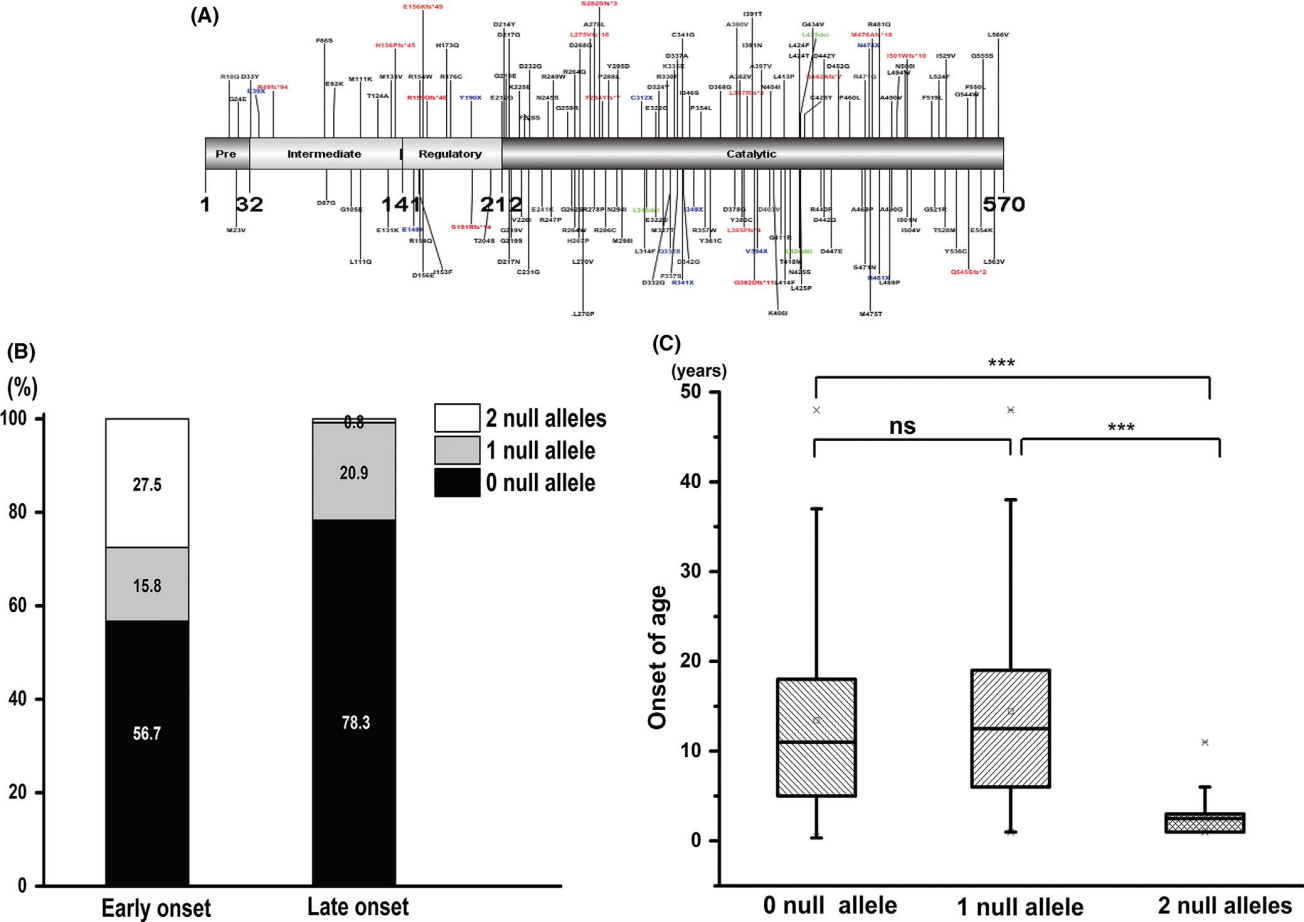
Genotype‐phenotype correlation. A, Location of the identified mutations in PANK2: PANK2 contains 570 amino acids. 141‐211aa represented the regulatory region, 212‐570aa represented the catalytic core region. Black, red, blue, and green represent missense mutation, frameshift mutation, nonsense mutation, and small fragment deletion, respectively. B, The proportion of patients carrying 0, 1, and 2 null alleles in early‐onset and late‐onset patients. In the early‐onset patients, 27.5% (33/120) carried two null alleles, and 15.8% (19/120) had one null allele, whereas, in the late‐onset patients, only 0.8% (1/115) carried two null alleles, and 20.9% (24/115) carried one null allele. C, The comparison of numbers of null alleles with the age of onset. The media age of disease onset in patients with 0 null allele, one null allele, and two null alleles was 11 y (0.33‐52.0), 13.0 y (1.0‐48.0) and 2.5 y (1.0‐11.0). Patients with two null alleles showed earlier age of disease onset. “**”denotes *P* < .05, “***” denotes *P* < .001, “ns” indicates no statistical difference

Among the 248 patients, 121 (48.8%) carried homozygous mutations of *PANK2*, and 114 (46.0%) carried compound heterozygous mutations. In 13 patients (13/248, 5.2%), mutation was detected only in one allele. A total of 176 mutations, including missense, frameshift, nonsense, small deletions, splice site mutations, and single or multiple exon deletion, were identified (Figure 3A). Most of the mutations were located in the core catalytic region of PANK2*.* c.1583C>T (p.T528M) was the most frequent mutation, which was identified in 23 patients.

We defined loss‐of‐function allele as “null allele,” which included nonsense, frameshift, +1 or +2 splicing mutations, −1 and −2 intronic mutations, and single or multiple exon deletion. In the 114 patients with compound heterozygous mutations, 37.8% (43/114) carried one null allele, and 1.8% (2/114) carried two null alleles. Of the 121 patients with homozygous mutations, 26.4% (32/121) carried null alleles.

To understand the genotype‐phenotype correlation, we analyzed the distribution of null allele in early‐onset patients and late‐onset patients. In the early‐onset patients, 27.5% (33/120) carried two null alleles, and 15.8% (19/120) had one null allele, whereas, in the late‐onset patients, only 0.8% (1/115) carried two null alleles, and 20.9% (24/115) carried one null allele. The proportion of the patients carrying two null alleles was significantly higher in early‐onset patients (*P* < .001) (Figure 3B). Genotype was significantly correlated with the age of onset. Patients with two null alleles showed earlier age of disease onset (*P* < .001), the media age of disease onset in patients with 0 null allele, one null allele, and two null alleles was 11 years (0.33‐52.0),13.0 years (1.0‐48.0), and 2.5 years (1.0‐11.0) (Figure 3C). Moreover, patients with two null alleles progressed more rapidly to loss of independent ambulance (*P* < .001).

### 3.4 Factors that influence disease progression

Multivariate logistic regression showed that patients with earlier age of onset (OR = 2.465, 95% CI: 1.359‐4.470, *P* = .003) and two null alleles (OR = 6.060, 95% CI: 1.954‐18.794, *P* = .002) progressed more rapidly to loss of independent ambulance.

## 4 DISCUSSION

PKAN is classified into classic and atypical type on the basis of both age of onset and disease progression. However, there are patients with early onset but slow progression or late onset with rapid progression, which were classified as “intermediate form” in some studies.^5^ In our study, patients were classified into early‐onset (before 10 years of age) and late‐onset (≥10 years of age), instead of classic and atypical types, to avoid the confusion of the “intermediate form.”

We conducted a detailed analysis of the natural history of PKAN on the basis of data from 248 patients. The median age at onset was 3.0 (0.3‐9.0) years in the early‐onset group and 18.0 (10.0‐52.0) years in the late‐onset group. Progression of dystonia was compared in two groups. Dystonia in lower limbs was the most common initial symptom in both early‐ and late‐onset groups. The proportion of patients who progressed to oromandibular dystonia, generalized dystonia, and even loss of independent ambulance was much higher in early‐onset patients. Early‐onset patients progressed more rapidly to loss of independent ambulance than late‐onset patients after disease onset. In patients who showed oromandibular dystonia, the occurrence of oromandibular dystonia in late‐onset patients was earlier after disease onset instead. In the early‐onset group, the median interval between the disease onset and occurrence of oromandibular dystonia, generalized dystonia, and loss of independent ambulance was 6.0 years (2.0‐13.0), 5.0 years (0.0‐16.0), and 5.0 years (0.0‐54.0), whereas the corresponding values in late‐onset group were 1.0 year (0.0‐22.0), 4.0 years (1.0‐11.0), and 6.0 years (0.0‐20.0). There were also differences in survival rates between the two groups. About 20.0% died at the median age of 12.5 (4.0‐24.0) years and 9.5 (2.0‐23.0) years after the onset in early‐onset group. Only 2.0% died in late‐onset patients during the follow‐up.

Brain function other than motor disability is less concerned in PKAN. We found that psychological and behavior problems were not uncommon. Compulsive behavior, emotional lability, anxiety, depression, or attention deficiency was reported in 44.9% (48/107) of early‐onset and 38.8% (38/98) of late‐onset patients. The cognitive decline in PKAN remains controversial because of the interference with the cognitive assessments due to severe motor handicap. About 54.0% (34/63) in early‐onset group and 33.8% (24/71) in late‐onset group were reported to show cognitive decline after disease onset. Freeman et al^6^ applied standardized scoring to evaluate the cognitive function of patients with PKAN, and 16 patients demonstrated different degrees of cognitive decline during the course. Mahoney et al^7^ found that the cognitive function of seven children who underwent deep brain stimulation (DBS) surgery improved with the improvement of dystonia, suggesting that severe motor dysfunction may affect the evaluation of cognitive function.

The “eye‐of‐the‐tiger” sign is the typical manifestation in neuroimaging, which is caused by the accumulation of iron in globus pallidus (abnormally low T2WI signal), tissue necrosis, and edema (high signal in T2WI). Seven out of eleven patients (63.6%) showed calcification in brain CT, which was probably associated with extracellular calcium deposits in globus pallidum.^8^ In contrast to idiopathic basal ganglia calcification, the calcification of patients with PKAN was limited to globus pallidus. However, CT was not considered a routine examination for patients suspected of PKAN; hence, the incidence of basal ganglion calcification might be underestimated.

As for the genotype‐phenotype correlation, we demonstrated that patients with two null alleles in *PANK2* showed significantly earlier age of disease onset and progressed more rapidly to loss of independent ambulance. The proportion of patients carrying two null alleles was significantly higher in the early‐onset patients than that in late‐onset patients. *PANK2* mutations likely decrease the function or abundance of PANK2 protein, thereby decreasing the production of coenzyme A, which plays an important role in ATP and fatty acid synthesis and in neurotransmitter metabolism. PANK2 dysfunction also results in the accumulation of cysteine and other substrates, possibly leading to oxidative stress injury of neurons. *PANK2* mutations are postulated to cause mitochondrial dysfunction because PANK2 enzyme localizes to the mitochondria in the human brain.^9^ The lower the activity of the enzyme residue, the earlier the age of onset in the previous study^10^; hence, residual enzyme activity was possibly lower in patients with two null alleles.

There is no curative treatment for PKAN yet. Only symptomatic treatments are available currently. Some treatments, such as deep brain stimulation, can improve the dystonia of patients with PKAN, but they do not modify the disease course. A randomized double‐blind controlled study about deferiprone for PKAN revealed that deferiprone can decrease the disease progression in patients with PKAN.^11^ Pantethine is used as a dietary supplement in some countries and administered to improve the exercise ability of PKAN mouse^12^ and *Drosophila* 13 models. However, whether pantethine is effective in patients with PKAN remains to be further confirmed. RE‐024 (Fosmetpantotenate) is a small molecule precursor of phosphopantothenic acid, a downstream product of pantothenic acid. In a case report y,^14^ RE‐024 could improve the symptoms of a patient with PKAN; however, in phase III clinical trial, there was no significant difference in primary endpoint between treatment groups and placebo groups according to Retrophin's announcement.

There are some limitations in this study. First, the original data were incomplete in some of the publications. Therefore, it was not possible to fully and comprehensively analyze the phenotype of the 248 patients. Second, most of studies were retrospective, which probably affect the accuracy of the data.

## 5 CONCLUSIONS

This study provided a comprehensive review on the natural history and genotype of 248 patients with PKAN. The time curve of progression in dystonia varied between the early‐onset and late‐onset patients. Behavior problems were not uncommon in both early‐ and late‐onset patients. Cognitive decline was reported, but interference of motor handicap on the assessments of cognitive function needs to be considered. A total of 176 mutations were summarized. We found that patients with two null alleles in *PANK2* showed significantly earlier age of disease onset. This suggested a correlation between the genotype and phenotype. The results of this study may serve as a historical control data for future clinical trial on PKAN.

## CONFLICT OF INTEREST

The authors declare no conflict of interest.

## ETHICAL STANDARD

This study was approved by the institutional review board of the ethics committee of Peking University First Hospital and has therefore been performed in accordance with the ethical standards laid down in the 1964 Declaration of Helsinki and its later amendments. The parents of all participants had been provided written informed consent for the use of the children's information for scientific purposes.
