# Supplementary material for: Natural history and genotype‐phenotype correlation of pantothenate kinase‐associated neurodegeneration
Source: CNS Neurosci Ther. 2020 Feb 11;26(7):754–61. doi: 10.1111/cns.13294 (PMC7298993; doi:10.1111/cns.13294)
Supplement: Supplementary file 1 [file CNS-26-754-s001.docx]

Natural history and genotype-phenotype correlation of pantothenate kinase associated neurodegeneration

Xuting Chang, Jie Zhang, Yuwu Jiang, Jingmin Wang, Ye Wu^*^

Department of Pediatrics, Peking University First Hospital, China

**Supplementary Information**

**Here lists the detailed information of 248 patients included in our study（Table 1）**

**Table1 Clinical, radiological, and genotypic characteristics of the patients studied**

| **ID** | **S** | **AA**  **LF**  **W** | **Age**  **At**  **onset** | **IS** | **Sur**  **vive** | | **Age**  **Of**  **death** | **Walk**  **Without**  **Support** | **Age**  **Of**  **LOA** | **OMD** | **Age**  **Of**  **OMD** | **LD** |  | **Age**  **Of**  **LD** | **GD** | **Age**  **Of**  **GD** | **dysphagia** | **Age**  **Of**  **dysphagia** | **dysarthria** | **Age of**  **dysarthria** | **PBD** | **DD** | **Pyramidal signs** | **Pigmentary retinopathy** | **EOTS（MRI）** | **GPC**  **(CT)** | **PANK2 mutation** | **Ref** |
| --- | --- | --- | --- | --- | --- | --- | --- | --- | --- | --- | --- | --- | --- | --- | --- | --- | --- | --- | --- | --- | --- | --- | --- | --- | --- | --- | --- | --- |
| **1** | **M** | **12** | **1** | **LLD** | | **Y** | **/** | **N** | **5** | **Y** | **8** | **Y** |  | **1** | **/** | **/** | **/** | **/** | **Y** | **1** | **N** | **/** | **Y** | **/** | **Y** | **/** | **c.628 + 2T > G**  **c.628 + 2T > G** | **[40]** |
| **2** | **F** | **12** | **1** | **LLD** | | **N** | **12** | **N** | **4** | **Y** | **8** | **Y** |  | **1** | **/** | **/** | **/** | **/** | **Y** | **1** | **N** | **/** | **Y** | **/** | **Y** | **/** | **c.628 + 2T > G**  **c.628 + 2T > G** | **[40]** |
| **3** | **F** | **9** | **1** | **VL** | | **Y** | **/** | **N** | **8** | **/** | **/** | **Y** |  | **2** | **/** | **/** | **/** | **/** | **Y** | **2** | **N** | **/** | **Y** | **/** | **Y** | **/** | **c.628 + 2T > G**  **c.628 + 2T > G** | **[40]** |
| **4** | **M** | **23** | **1** | **LLD** | | **N** | **23** | **N** | **22** | **Y** | **12** | **Y** |  | **1** | **/** | **/** | **/** | **/** | **Y** | **1** | **N** | **/** | **Y** | **/** | **Y** | **/** | **c.994C＞T**  **c.994C＞T** | **[40]** |
| **5** | **M** | **19** | **1** | **LLD** | | **N** | **19** | **N** | **8** | **Y** | **14** | **Y** |  | **1** | **/** | **/** | **/** | **/** | **Y** | **1** | **N** | **/** | **Y** | **/** | **Y** | **/** | **c.994C＞T**  **c.994C＞T** | **[40]** |
| **6** | **F** | **13** | **2** | **LLD** | | **Y** | **/** | **N** | **5** | **Y** | **4** | **Y** |  | **2** | **/** | **/** | **/** | **/** | **Y** | **2** | **N** | **/** | **Y** | **/** | **Y** | **/** | **c.994C＞T**  **c.994C＞T** | **[40]** |
| **7** | **M** | **11** | **1** | **LLD** | | **Y** | **/** | **N** | **6** | **Y** | **10** | **Y** |  | **1** | **/** | **/** | **/** | **/** | **/** | **/** | **N** | **/** | **Y** | **/** | **Y** | **/** | **c.13251328delATAG** | **[40]** |
| **8** | **F** | **6** | **1** | **LLD** | | **Y** | **/** | **Y** | **/** | **Y** | **5** | **Y** |  | **1** | **/** | **/** | **/** | **/** | **Y** | **1** | **N** | **/** | **Y** | **/** | **Y** | **/** | **c.1537-2A > G** | **[40]** |
| **9** | **F** | **24** | **1** | **LLD** | | **N** | **24** | **N** | **7** | **Y** | **7** | **Y** |  | **1** | **/** | **/** | **/** | **/** | **Y** | **1** | **N** | **/** | **Y** | **/** | **Y** | **/** | **c.1481T > G**  **c.1481T > G** | **[40]** |
| **10** | **M** | **21** | **8** | **VL** | | **Y** | **/** | **N** | **12** | **Y** | **14** | **Y** |  | **10** | **/** | **/** | **/** | **/** | **Y** | **12** | **N** | **/** | **N** | **/** | **Y** | **/** | **c.1660G > A**  **c.1660G > A** | **[40]** |
| **11** | **M** | **25** | **8** | **VL** | | **Y** | **/** | **N** | **11** | **Y** | **17** | **Y** |  | **10** | **/** | **/** | **/** | **/** | **Y** | **9** | **N** | **/** | **N** | **/** | **Y** | **/** | **c.1660G > A**  **c.1660G > A** | **[40]** |
| **12** | **M** | **14** | **7** | **VL** | | **Y** | **/** | **Y** | **/** | **Y** | **9** | **Y** |  | **13** | **/** | **/** | **/** | **/** | **Y** | **10** | **N** | **/** | **N** | **/** | **Y** | **/** | **c.1660G > A**  **c.1660G > A** | **[40]** |
| **13** | **M** | **25** | **5** | **VL** | | **Y** | **/** | **Y** | **/** | **/** | **/** | **/** |  | **/** | **/** | **/** | **/** | **/** | **Y** | **21** | **Y** | **/** | **N** | **/** | **Y** | **/** | **c.655G > A**  **c.655G > A** | **[40]** |
| **14** | **F** | **39** | **4** | **VL** | | **Y** | **/** | **Y** | **/** | **/** | **/** | **Y** |  | **28** | **/** | **/** | **/** | **/** | **Y** | **21** | **Y** | **/** | **N** | **/** | **Y** | **/** | **c.655G > A**  **c.655G > A** | **[40]** |
| **15** | **M** | **23** | **8** | **VL** | | **Y** | **/** | **Y** | **/** | **/** | **/** | **/** |  | **/** | **/** | **/** | **/** | **/** | **/** | **/** | **Y** | **/** | **N** | **/** | **Y** | **/** | **c.655G > A**  **c.655G > A** | **[40]** |
| **16** | **F** | **21** | **5** | **VL** | | **Y** | **/** | **Y** | **/** | **/** | **/** | **/** |  | **/** | **/** | **/** | **/** | **/** | **/** | **/** | **Y** | **/** | **N** | **/** | **Y** | **/** | **c.655G > A**  **c.655G > A** | **[40]** |
| **17** | **F** | **20** | **12** | **LLD** | | **Y** | **/** | **N** | **18** | **Y** | **18** | **Y** |  | **12** | **/** | **/** | **/** | **/** | **/** | **/** | **N** | **/** | **Y** | **/** | **Y** | **/** | **c.676G > A**  **c.676G > A** | **[40]** |
| **18** | **F** | **20** | **16** | **LLD** | | **Y** | **/** | **Y** | **/** | **/** | **/** | **Y** |  | **16** | **/** | **/** | **/** | **/** | **Y** | **18** | **Y** | **/** | **N** | **/** | **Y** | **/** | **c.676G > A**  **c.676G > A** | **[40]** |
| **19** | **F** | **14** | **12** | **LLD** | | **Y** | **/** | **Y** | **/** | **/** | **/** | **Y** |  | **12** | **/** | **/** | **/** | **/** | **/** | **/** | **Y** | **/** | **N** | **/** | **Y** | **/** | **c.676G > A**  **c.676G > A** | **[40]** |
| **20** | **F** | **22** | **15** | **LLD** | | **Y** | **/** | **Y** | **/** | **/** | **/** | **Y** |  | **15** | **/** | **/** | **/** | **/** | **/** | **/** | **N** | **/** | **N** | **/** | **Y** | **/** | **c.800A > C**  **c.800A > C** | **[40]** |
| **21** | **F** | **23** | **15** | **LLD** | | **Y** | **/** | **N** | **23** | **Y** | **20** | **Y** |  | **15** | **/** | **/** | **/** | **/** | **Y** | **15** | **N** | **/** | **N** | **/** | **Y** | **/** | **c.833G > T**  **c.833G > T** | **[40]** |
| **22** | **M** | **37** | **16** | **OMD** | | **Y** | **/** | **N** | **24** | **Y** | **16** | **/** |  | **/** | **Y** | **22** | **/** | **/** | **/** | **/** | **Y** | **/** | **Y** | **/** | **Y** | **/** | **c.1466T > C**  **c.1583C > T** | **[40]** |
| **23** | **F** | **14** | **8** | **LLD** | | **N** | **14** | **N** | **12** | **Y** | **/** | **Y** |  | **8** | **Y** | **/** | **Y** | **/** | **Y** | **/** | **/** | **N** | **Y** | **Y** | **/** | **/** | **c.1171_1174dupAT c.1171_1174dupAT** | **[65]** |
| **24** | **M** | **36** | **18** | **PS** | | **Y** | **/** | **Y** | **/** | **/** | **/** | **N** |  | **/** | **/** | **/** | **N** | **/** | **N** | **/** | **N** | **/** | **N** | **/** | **Y** | **/** | **c.1408insT** | **[24]** |
| **25** | **M** | **24** | **10** | **LLD** | | **N** | **24** | **N** | **22** | **/** | **/** | **Y** |  | **10** | **Y** | **/** | **/** | **/** | **Y** | **10** | **N** | **N** | **/** | **/** | **Y** | **N** | **c.1391C＞T**  **c.1391C＞T** | **[50]** |
| **26** | **M** | **12** | **1** | **DD** | | **Y** | **/** | **N** | **10** | **/** | **/** | **Y** |  | **/** | **Y** | **/** | **/** | **/** | **Y** | **/** | **N** | **Y** | **Y** | **Y** | **Y** | **/** | **c.883 T＞G**  **c.883 T＞G** | **[16]** |
| **27** | **F** | **19** | **6** | **ULD** | | **Y** | **/** | **N** | **10** | **/** | **/** | **Y** |  | **6** | **Y** | **/** | **/** | **/** | **Y** | **/** | **Y** | **N** | **N** | **N** | **Y** | **/** | **c.740G＞C**  **c.1231G＞A** | **[16]** |
| **28** | **F** | **13** | **10** | **ULD** | | **Y** | **/** | **N** | **10** | **/** | **/** | **Y** |  | **10** | **Y** | **/** | **/** | **/** | **Y** | **/** | **N** | **/** | **N** | **N** | **Y** | **/** | **c.1231G＞A**  **c.1253C＞T** | **[16]** |
| **29** | **M** | **14** | **1** | **ULD** | | **Y** | **/** | **N** | **9** | **/** | **/** | **Y** |  | **1** | **/** | **/** | **/** | **/** | **Y** | **/** | **N** | **/** | **Y** | **N** | **Y** | **/** | **c.493_494del** | **[16]** |
| **30** | **F** | **14** | **9** | **LLD** | | **Y** | **/** | **N** | **9** | **/** | **/** | **Y** |  | **9** | **Y** | **/** | **/** | **/** | **Y** | **/** | **N** | **/** | **Y** | **N** | **Y** | **/** | **c.1231G＞A** | **[16]** |
| **31** | **M** | **26** | **11** | **PBD** | | **Y** | **/** | **Y** | **/** | **/** | **/** | **Y** |  | **/** | **Y** | **/** | **/** | **/** | **Y** | **/** | **Y** | **/** | **Y** | **N** | **Y** | **/** | **c.460C＞T**  **c.635A＞G** | **[16]** |
| **32** | **M** | **51** | **12** | **LLD** | | **Y** | **/** | **Y** | **/** | **/** | **/** | **Y** |  | **12** | **Y** | **/** | **/** | **/** | **Y** | **/** | **N** | **/** | **Y** | **N** | **Y** | **/** | **c.526C＞T**  **c.526C＞T** | **[16]** |
| **33** | **F** | **43** | **12** | **LLD** | | **Y** | **/** | **Y** | **/** | **/** | **/** | **Y** |  | **12** | **N** | **/** | **/** | **/** | **Y** | **/** | **N** | **/** | **Y** | **N** | **Y** | **/** | **c.526C＞T**  **c.526C＞T** | **[16]** |
| **34** | **M** | **51** | **12** | **BD** | | **Y** | **/** | **Y** | **/** | **/** | **/** | **Y** |  | **/** | **Y** | **/** | **/** | **/** | **Y** | **/** | **Y** | **/** | **Y** | **Y** | **Y** | **/** | **c.740G＞C**  **c.740G＞C** | **[16]** |
| **35** | **F** | **22** | **2** | **LLD** | | **Y** | **/** | **Y** | **/** | **Y** | **/** | **N** |  | **/** | **/** | **/** | **/** | **/** | **Y** | **/** | **Y** | **N** | **Y** | **N** | **Y** | **/** | **IVS1+5G＞C**  **c.635A＞G** | **[16]** |
| **36** | **F** | **42** | **5** | **LLD** | | **Y** | **/** | **N** | **32** | **/** | **/** | **Y** |  | **5** | **Y** | **/** | **/** | **/** | **Y** | **/** | **Y** | **/** | **Y** | **Y** | **Y** | **/** | **c.850 851del**  **c.461G＞A** | **[16]** |
| **37** | **M** | **23** | **5** | **PBD** | | **Y** | **/** | **Y** | **/** | **/** | **/** | **Y** |  | **/** | **Y** | **/** | **/** | **/** | **Y** | **/** | **Y** | **/** | **Y** | **N** | **Y** | **/** | **c.526C＞T**  **c.526C＞T** | **[16]** |
| **38** | **M** | **42** | **17** | **ULD** | | **Y** | **/** | **N** | **29** | **/** | **/** | **Y** |  | **17** | **Y** | **/** | **/** | **/** | **Y** | **/** | **Y** | **/** | **Y** | **N** | **Y** | **/** | **c.740G＞C**  **c.740G＞C** | **[16]** |
| **39** | **F** | **42** | **5** | **DD** | | **Y** | **/** | **Y** | **/** | **/** | **/** | **Y** |  | **/** | **N** | **/** | **/** | **/** | **Y** | **/** | **N** | **/** | **Y** | **N** | **Y** | **/** | **c.1021C＞T**  **c.1231G＞A** | **[16]** |
| **40** | **M** | **20** | **10** | **LLD** | | **Y** | **/** | **Y** | **/** | **/** | **/** | **Y** |  | **10** | **Y** | **/** | **/** | **/** | **Y** | **/** | **Y** | **/** | **Y** | **N** | **Y** | **/** | **c.526C＞T**  **c.526C＞T** | **[16]** |
| **41** | **M** | **22** | **14** | **ULD** | | **Y** | **/** | **Y** | **/** | **/** | **/** | **Y** |  | **14** | **Y** | **/** | **/** | **/** | **Y** | **/** | **N** | **/** | **Y** | **N** | **Y** | **/** | **c.1231G＞A**  **c.1253C＞T** | **[16]** |
| **42** | **F** | **13** | **10** | **LLD** | | **Y** | **/** | **Y** | **/** | **/** | **/** | **Y** |  | **10** | **/** | **/** | **/** | **/** | **/** | **/** | **Y** | **N** | **N** | **N** | **N** | **/** | **IVS c.540-13_540-12insTTCCCC** | **[16]** |
| **43** | **M** | **15** | **3** | **ULD** | | **Y** | **/** | **N** | **12** | **Y** | **/** | **Y** |  | **3** | **Y** | **/** | **Y** | **/** | **Y** | **/** | **Y** | **N** | **N** | **Y** | **Y** | **/** | **c.1171_1174dupAT c.1171_1174dupAT** | **[65]** |
| **44** | **F** | **4** | **3** | **LLD** | | **Y** | **/** | **Y** | **/** | **N** | **/** | **Y** |  | **5** | **N** | **/** | **Y** | **/** | **N** | **/** | **Y** | **N** | **Y** | **Y** | **Y** | **/** | **c.1171_1174dupAT c.1171_1174dupAT** | **[65]** |
| **45** | **F** | **/** | **1** | **LLD** | | **/** | **/** | **N** | **6** | **Y** | **/** | **Y** |  | **1** | **Y** | **/** | **/** | **/** | **/** | **/** | **Y** | **/** | **Y** | **N** | **Y** | **/** | **del exon 3 +4**  **del exon 3 +4** | **[28]** |
| **46** | **F** | **/** | **1** | **LLD** | | **/** | **/** | **N** | **＞5** | **Y** | **/** | **Y** |  | **1** | **Y** | **/** | **/** | **/** | **/** | **/** | **Y** | **/** | **Y** | **N** | **Y** | **/** | **c.821_822del**  **del exon 1–4** | **[28]** |
| **47** | **M** | **/** | **2** | **GD** | | **/** | **/** | **N** | **2** | **Y** | **/** | **/** |  | **/** | **Y** | **2** | **/** | **/** | **/** | **/** | **Y** | **/** | **Y** | **N** | **Y** | **/** | **c.1442_1444del**  **c.1583C＞T** | **[28]** |
| **48** | **F** | **/** | **2** | **GD** | | **/** | **/** | **N** | **4** | **Y** | **/** | **/** |  | **/** | **Y** | **2** | **/** | **/** | **Y** | **/** | **Y** | **/** | **Y** | **Y** | **Y** | **/** | **c.1561G＞A**  **c.573delC** | **[28]** |
| **49** | **F** | **/** | **2** | **GD** | | **/** | **/** | **N** | **5** | **Y** | **/** | **/** |  | **/** | **Y** | **2** | **/** | **/** | **Y** | **/** | **Y** | **/** | **Y** | **Y** | **Y** | **/** | **c.1561G＞A**  **c.573delC** | **[28]** |
| **50** | **F** | **/** | **2** | **LLD** | | **/** | **/** | **N** | **16** | **Y** | **/** | **Y** |  | **2** | **Y** | **/** | **/** | **/** | **/** | **/** | **Y** | **/** | **Y** | **Y** | **Y** | **/** | **c.569dupA**  **c.569dupA** | **[28]** |
| **51** | **F** | **/** | **2** | **LLD** | | **/** | **/** | **N** | **20** | **Y** | **/** | **Y** |  | **2** | **Y** | **/** | **/** | **/** | **/** | **/** | **Y** | **/** | **Y** | **Y** | **Y** | **/** | **c.683T＞C**  **c.1648T＞C** | **[28]** |
| **52** | **F** | **/** | **2** | **dystaxia** | | **/** | **/** | **N** | **＞6** | **Y** | **/** | **/** |  | **/** | **/** | **/** | **/** | **/** | **Y** | **/** | **Y** | **/** | **Y** | **/** | **Y** | **/** | **c.821_822del**  **c.1561G ＞A** | **[28]** |
| **53** | **M** | **/** | **3** | **GD** | | **/** | **/** | **N** | **6** | **Y** | **/** | **/** |  | **/** | **Y** | **/** | **/** | **/** | **Y** | **/** | **Y** | **/** | **Y** | **Y** | **Y** | **/** | **c.1561G＞A**  **c.1561G＞A** | **[28]** |
| **54** | **F** | **/** | **3** | **PS** | | **/** | **/** | **N** | **6** | **Y** | **/** | **Y** |  | **3** | **Y** | **/** | **/** | **/** | **Y** | **/** | **Y** | **/** | **Y** | **Y** | **Y** | **/** | **c.1323_1326del**  **c.1323_1326del** | **[28]** |
|  |  |  |  |  | |  |  |  |  |  |  |  |  |  |  |  |  |  |  |  |  |  |  |  |  |  |  |  |
| **55** | **F** | **/** | **3** | **GD** | | **/** | **/** | **N** | **11** | **Y** | **/** | **/** |  | **/** | **Y** | **/** | **/** | **/** | **Y** | **/** | **Y** | **/** | **Y** | **Y** | **Y** | **/** | **c.573delC**  **c.573delC** | **[28]** |
| **56** | **M** | **/** | **3** | **dystaxia** | | **/** | **/** | **N** | **3** | **N** | **/** | **/** |  | **/** | **N** | **/** | **/** | **/** | **/** | **/** | **N** | **/** | **Y** | **N** | **Y** | **/** | **c.1663-1G＞C**  **del exon 5** | **[28]** |
| **57** | **F** | **/** | **3** | **GD** | | **/** | **/** | **N** | **5** | **Y** | **/** | **/** |  | **/** | **Y** | **/** | **/** | **/** | **Y** | **/** | **Y** | **/** | **Y** | **N** | **Y** | **/** | **c.573delC**  **c.573delC** | **[28]** |
| **58** | **F** | **/** | **3** | **GD** | | **/** | **/** | **N** | **7** | **Y** | **/** | **/** |  | **/** | **Y** | **/** | **/** | **/** | **Y** | **/** | **Y** | **/** | **Y** | **N** | **Y** | **/** | **c.994C＞T**  **c.994C＞T** | **[28]** |
| **59** | **M** | **/** | **4** | **GD** | | **/** | **/** | **N** | **7** | **Y** | **/** | **/** |  | **/** | **Y** | **/** | **/** | **/** | **Y** | **/** | **Y** | **/** | **Y** | **Y** | **Y** | **/** | **c.1274T＞C**  **c.1274T＞C** | **[28]** |
| **60** | **M** | **/** | **4** | **LLD** | | **/** | **/** | **N** | **8** | **Y** | **/** | **Y** |  | **4** | **Y** | **/** | **/** | **/** | **/** | **/** | **Y** | **/** | **Y** | **N** | **Y** | **/** | **c.1499A＞T**  **c.1499A＞T** | **[28]** |
| **61** | **F** | **/** | **4** | **ULD** | | **/** | **/** | **N** | **8** | **N** | **/** | **Y** |  | **4** | **Y** | **/** | **/** | **/** | **Y** | **/** | **N** | **/** | **N** | **N** | **Y** | **/** | **c.894G＞A**  **c.1418–1424del** | **[28]** |
| **62** | **M** | **/** | **4** | **LLD** | | **/** | **/** | **N** | **19** | **Y** | **/** | **Y** |  | **4** | **Y** | **/** | **/** | **/** | **/** | **/** | **Y** | **/** | **Y** | **Y** | **Y** | **/** | **c.790C＞T**  **c.1412G＞A** | **[28]** |
| **63** | **M** | **/** | **4** | **GD** | | **/** | **/** | **N** | **7** | **Y** | **/** | **/** |  | **/** | **Y** | **4** | **/** | **/** | **/** | **/** | **Y** | **/** | **Y** | **Y** | **Y** | **/** | **c.573delC**  **c.1274T＞C** | **[28]** |
| **64** | **F** | **/** | **5** | **LLD** | | **/** | **/** | **N** | **13** | **Y** | **/** | **/** |  | **/** | **Y** | **/** | **/** | **/** | **/** | **/** | **N** | **/** | **Y** | **Y** | **Y** | **/** | **c.649G＞A**  **c.649G＞A** | **[28]** |
| **65** | **F** | **/** | **6** | **GD** | | **/** | **/** | **N** | **9** | **Y** | **/** | **/** |  | **/** | **Y** | **/** | **/** | **/** | **Y** | **/** | **Y** | **/** | **Y** | **Y** | **Y** | **/** | **c.1561G＞A**  **c.1632dupG** | **[28]** |
| **66** | **M** | **/** | **6** | **GD** | | **/** | **/** | **N** | **9** | **Y** | **/** | **/** |  | **/** | **Y** | **6** | **/** | **/** | **Y** | **/** | **Y** | **/** | **Y** | **Y** | **Y** | **/** | **c.573delC**  **c.573delc** | **[28]** |
| **67** | **M** | **/** | **7** | **LLD** | | **/** | **/** | **N** | **15** | **Y** | **/** | **Y** |  | **7** | **Y** | **/** | **/** | **/** | **Y** | **/** | **Y** | **/** | **Y** | **Y** | **Y** | **/** | **c.856C＞T**  **c.1561G＞A** | **[28]** |
| **68** | **F** | **/** | **6** | **LLD** | | **/** | **/** | **N** | **60** | **N** | **/** | **Y** |  | **6** | **Y** | **/** | **/** | **/** | **/** | **/** | **Y** | **/** | **/** | **/** | **Y** | **/** | **c.1561G＞A**  **c.1442G＞A** | **[28]** |
| **69** | **M** | **/** | **7** | **dystonia** | | **/** | **/** | **N** | **10** | **Y** | **/** | **/** |  | **/** | **Y** | **/** | **/** | **/** | **Y** | **/** | **Y** | **/** | **Y** | **N** | **Y** | **/** | **c.573delC**  **c.1583C＞T** | **[28]** |
| **70** | **F** | **/** | **8** | **GD** | | **/** | **/** | **N** | **9** | **N** | **/** | **/** |  | **/** | **Y** | **/** | **/** | **/** | **Y** | **/** | **Y** | **/** | **Y** | **Y** | **Y** | **/** | **c.1274T＞C**  **c.1283G＞ A** | **[28]** |
| **71** | **M** | **/** | **8** | **LLD** | | **/** | **/** | **N** | **21** | **N** | **/** | **/** |  | **/** | **Y** | **/** | **/** | **/** | **/** | **/** | **Y** | **/** | **Y** | **Y** | **Y** | **/** | **c.1238T＞C**  **c.965A＞G** | **[28]** |
| **72** | **M** | **/** | **10** | **PBD** | | **/** | **/** | **N** | **18** | **Y** | **/** | **/** |  | **/** | **Y** | **/** | **/** | **/** | **/** | **/** | **Y** | **/** | **Y** | **Y** | **Y** | **/** | **c.790C＞T**  **c.790C＞T** | **[28]** |
| **73** | **M** | **/** | **10** | **PBD** | | **/** | **/** | **N** | **27** | **Y** | **/** | **/** |  | **/** | **Y** | **/** | **/** | **/** | **/** | **/** | **Y** | **/** | **Y** | **/** | **Y** | **/** | **c.790C＞T**  **c.965A＞G** | **[28]** |
| **74** | **F** | **/** | **12** | **LLD** | | **/** | **/** | **N** | **30** | **Y** | **/** | **Y** |  | **12** | **Y** | **/** | **/** | **/** | **/** | **/** | **Y** | **/** | **Y** | **Y** | **Y** | **/** | **c.1176_1177del**  **c.791G＞A** | **[28]** |
| **75** | **F** | **/** | **13** | **OMD** | | **/** | **/** | **N** | **27** | **Y** | **13** | **/** |  | **/** | **Y** | **/** | **/** | **/** | **Y** | **/** | **Y** | **/** | **N** | **N** | **Y** | **/** | **c.1418_1424del**  **c.1583C＞T** | **[28]** |
| **76** | **M** | **/** | **14** | **Dysarthria** | | **/** | **/** | **N** | **19** | **Y** | **/** | **/** |  | **/** | **Y** | **/** | **/** | **/** | **Y** | **14** | **Y** | **/** | **N** | **N** | **Y** | **/** | **c.1561G＞A**  **c.1583C＞T** | **[28]** |
| **77** | **F** | **/** | **14** | **PBD** | | **/** | **/** | **N** | **17** | **Y** | **/** | **/** |  | **/** | **Y** | **/** | **/** | **/** | **/** | **/** | **Y** | **/** | **Y** | **Y** | **Y** | **/** | **c.1069C＞T**  **c.1069C＞T** | **[28]** |
| **78** | **M** | **/** | **17** | **OMD** | | **/** | **/** | **N** | **17** | **Y** | **17** | **/** |  | **/** | **Y** | **/** | **/** | **/** | **Y** | **/** | **Y** | **/** | **Y** | **N** | **Y** | **/** | **c.1583C＞T**  **c.1583C＞T** | **[28]** |
| **79** | **F** | **/** | **17** | **LLD** | | **/** | **/** | **N** | **23** | **Y** | **/** | **Y** |  | **17** | **Y** | **/** | **/** | **/** | **Y** | **/** | **N** | **/** | **Y** | **/** | **Y** | **/** | **c.790C ＞T**  **c.856C ＞T** | **[28]** |
| **80** | **M** | **/** | **18** | **LLD** | | **/** | **/** | **N** | **21** | **Y** | **/** | **Y** |  | **18** | **Y** | **/** | **/** | **/** | **/** | **/** | **N** | **/** | **Y** | **N** | **Y** | **/** | **c.1441C＞T**  **c.966G＞T** | **[28]** |
| **81** | **M** | **/** | **18** | **ULD** | | **/** | **/** | **N** | **＞23** | **Y** | **/** | **Y** |  | **18** | **/** | **/** | **/** | **/** | **/** | **/** | **N** | **/** | **Y** | **N** | **Y** | **/** | **c.856C＞T**  **c.856C＞T** | **[28]** |
| **82** | **F** | **/** | **19** | **OMD** | | **/** | **/** | **N** | **34** | **Y** | **19** | **/** |  | **/** | **Y** | **/** | **/** | **/** | **Y** | **/** | **Y** | **/** | **Y** | **N** | **Y** | **/** | **c.1418_1424del**  **c.1583C＞T** | **[28]** |
| **83** | **F** | **/** | **20** | **LLD** | | **/** | **/** | **N** | **20** | **Y** | **/** | **Y** |  | **20** | **Y** | **/** | **/** | **/** | **Y** | **/** | **Y** | **/** | **Y** | **N** | **Y** | **/** | **c.1583C＞T**  **c.1583C＞T** | **[28]** |
| **84** | **F** | **/** | **25** | **OMD** | | **/** | **/** | **N** | **＞23** | **Y** | **25** | **/** |  | **/** | **Y** | **/** | **/** | **/** | **Y** | **/** | **Y** | **/** | **Y** | **N** | **Y** | **/** | **c.1583C＞T**  **c.1583C＞T** | **[28]** |
| **85** | **M** | **/** | **30** | **PBD** | | **/** | **/** | **N** | **＞30** | **Y** | **30** | **/** |  | **/** | **Y** | **/** | **/** | **/** | **Y** | **/** | **Y** | **/** | **Y** | **N** | **Y** | **/** | **c.1441C＞T**  **c.775G＞A** | **[28]** |
| **86** | **F** | **13** | **10** | **LLD** | | **/** | **/** | **/** | **/** | **/** | **/** | **Y** |  | **10** | **N** | **/** | **N** | **/** | **N** | **/** | **Y** | **/** | **/** | **N** | **N** | **/** | **IVSc.540-13_540-12insTTCCCC** | **[29]** |
| **87** | **F** | **24** | **18** | **PS** | | **Y** | **/** | **Y** | **/** | **N** | **/** | **Y** |  | **21** | **N** | **/** | **N** | **/** | **N** | **/** | **N** | **N** | **N** | **N** | **Y** | **/** | **c.764＞G**  **c.764A＞G** | **[60]** |
| **88** | **F** | **9** | **4** | **fracture** | | **N** | **9** | **N** | **/** | **N** | **/** | **Y** |  | **8** | **Y** | **/** | **/** | **/** | **Y** | **/** | **N** | **/** | **/** | **/** | **Y** | **/** | **c.1426_1429delATGc.1426_1429delATGA** | **[34]** |
| **89** | **M** | **12** | **6** | **LLD** | | **Y** | **/** | **N** | **10** | **Y** | **/** | **Y** |  | **6** | **Y** | **8** | **Y** | **/** | **N** | **/** | **/** | **N** | **Y** | **Y** | **Y** | **/** | **c.1171_1174dupAT**  **c.519C>G** | **[65]** |
| **90** | **M** | **55** | **25** | **PS** | | **Y** | **/** | **Y** | **/** | **/** | **/** | **Y** |  | **25** | **/** | **/** | **/** | **/** | **/** | **/** | **N** | **N** | **/** | **/** | **Y** | **/** | **c.1069C＞T**  **c.1069C＞T** | **[5]** |
| **91** | **F** | **52** | **22** | **PS** | | **Y** | **/** | **Y** | **/** | **/** | **/** | **Y** |  | **22** | **/** | **/** | **/** | **/** | **/** | **/** | **N** | **N** | **/** | **/** | **Y** | **/** | **c.1069C＞T**  **c.1069C＞T** | **[5]** |
| **92** | **F** | **51** | **48** | **Dysarthria** | | **Y** | **/** | **Y** | **/** | **N** | **/** | **N** |  | **/** | **N** | **/** | **Y** | **48** | **Y** | **48** | **N** | **N** | **/** | **/** | **Y** | **/** | **c.881A＞T**  **c..881A＞T** | **[33]** |
| **93** | **F** | **5** | **1** | **DD** | | **Y** | **/** | **Y** | **/** | **N** | **/** | **Y** |  | **4** | **N** | **/** | **/** | **/** | **/** | **/** | **/** | **Y** | **/** | **/** | **Y** | **/** | **c.683T＞C**  **c.1561G＞A** | **[30]** |
| **94** | **M** | **12** | **11.5** | **LD** | | **Y** | **/** | **Y** | **/** | **N** | **/** | **Y** |  | **11.5** | **N** | **/** | **/** | **/** | **N** | **/** | **N** | **N** | **N** | **N** | **Y** | **/** | **c,1555T＞C**  **c.1670A＞G** | **[56]** |
| **95** | **F** | **18** | **14** | **IM** | | **Y** | **/** | **Y** | **/** | **Y** | **/** | **Y** |  | **14** | **Y** | **/** | **/** | **/** | **Y** | **18** | **Y** | **N** | **/** | **/** | **N** | **Y** | **c.1583C＞T**  **c.1561G＞A** | **[61]** |
| **96** | **F** | **20** | **12** | **LLD** | | **Y** | **/** | **Y** | **/** | **N** | **/** | **Y** |  | **12** | **N** | **/** | **N** | **/** | **Y** | **14** | **N** | **N** | **N** | **/** | **Y** | **/** | **c.863C＞T**  **c.863C＞T** | **[22]** |
| **97** | **F** | **21** | **14** | **PBD** | | **Y** | **/** | **Y** | **/** | **N** | **/** | **Y** |  | **21** | **N** | **/** | **N** | **/** | **Y** | **21** | **Y** | **N** | **Y** | **N** | **Y** | **/** | **c.1573-3C＞G** | **[53]** |
| **98** | **F** | **11** | **2** | **LLD** | | **Y** | **/** | **N** | **11** | **/** | **/** | **Y** |  | **2** | **Y** | **/** | **Y** | **/** | **Y** | **/** | **/** | **Y** | **Y** | **/** | **Y** | **/** | **c.936T＞A**  **c.936T＞A** | **[49]** |
| **99** | **F** | **1.8** | **1** | **DD** | | **Y** | **/** | **N** | **/** | **N** | **/** | **N** |  | **/** | **N** | **/** | **N** | **/** | **N** | **/** | **N** | **Y** | **N** | **/** | **N** | **/** | **c.936T＞A**  **c.936T＞A** | **[49]** |
| **100** | **F** | **12** | **6** | **dystonia** | | **N** | **12** | **N** | **/** | **Y** | **/** | **Y** |  | **/** | **Y** | **/** | **Y** | **/** | **N** | **/** | **N** | **Y** | **/** | **Y** | **/** | **/** | **c.1231G＞A**  **del exon1** | **[26]** |
| **101** | **M** | **20** | **0.5** | **DD** | | **N** | **20** | **/** | **/** | **Y** | **10** | **Y** |  | **10** | **Y** | **10** | **Y** | **/** | **/** | **/** | **/** | **Y** | **Y** | **Y** | **/** | **/** | **c.1231G＞A**  **c.1231G＞A** | **[26]** |
| **102** | **M** | **6** | **4.5** | **IM** | | **Y** | **/** | **N** | **6** | **/** | **/** | **Y** |  | **/** | **Y** | **/** | **Y** | **6** | **Y** | **6** | **N** | **Y** | **Y** | **N** | **Y** | **Y** | **c.650A＞G**  **c.1341T＞G** | **[11]** |
| **103** | **M** | **21** | **10** | **LD** | | **Y** | **/** | **/** | **/** | **Y** | **/** | **Y** |  | **10** | **Y** | **/** | **/** | **/** | **Y** | **/** | **N** | **/** | **N** | **N** | **Y** | **/** | **c.1405G＞C**  **c.1211A＞T** | **[17]** |
| **104** | **F** | **16** | **16** | **ULD** | | **Y** | **/** | **/** | **/** | **Y** | **/** | **Y** |  | **16** | **N** | **/** | **/** | **/** | **Y** | **/** | **Y** | **/** | **N** | **N** | **Y** | **/** | **c.1405G＞C**  **c.1211A＞T** | **[17]** |
| **105** | **M** | **30** | **26** | **Blepharospasm** | | **Y** | **/** | **/** | **/** | **Y** | **/** | **Y** |  | **/** | **Y** | **/** | **/** | **/** | **Y** | **/** | **Y** | **/** | **Y** | **N** | **Y** | **/** | **c.1211A＞T**  **c.1211A＞T** | **[17]** |
| **106** | **F** | **45** | **3** | **LLD** | | **Y** | **/** | **/** | **/** | **N** | **/** | **Y** |  | **3** | **N** | **/** | **/** | **/** | **Y** | **/** | **Y** | **/** | **Y** | **N** | **Y** | **/** | **c.1211A＞T**  **c.1211A＞T** | **[17]** |
| **107** | **M** | **55** | **25** | **LLD** | | **Y** | **/** | **/** | **/** | **N** | **/** | **Y** |  | **25** | **N** | **/** | **/** | **/** | **Y** | **/** | **Y** | **/** | **Y** | **N** | **Y** | **/** | **c.1211A＞T**  **c.1211A＞T** | **[17]** |
| **109** | **M** | **42** | **18** | **LLD** | | **Y** | **/** | **/** | **/** | **N** | **/** | **Y** |  | **18** | **N** | **/** | **/** | **/** | **Y** | **/** | **Y** | **/** | **N** | **N** | **Y** | **/** | **c.1211A＞T**  **c.1211A＞T** | **[17]** |
| **108** | **M** | **49** | **8** | **Dysarthria** | | **Y** | **/** | **/** | **/** | **Y** | **/** | **N** |  | **/** | **N** | **/** | **/** | **/** | **Y** | **8** | **Y** | **/** | **Y** | **N** | **Y** | **/** | **c.1211A＞T**  **c.1211A＞T** | **[17]** |
| **110** | **F** | **37** | **18** | **PBD** | | **Y** | **/** | **/** | **/** | **N** | **/** | **N** |  | **/** | **N** | **/** | **/** | **/** | **Y** |  | **Y** | **/** | **N** | **N** | **Y** | **/** | **c.1211A＞T**  **c.1211A＞T** | **[17]** |
| **111** | **F** | **9** | **5** | **ULD** | | **N** | **9** | **/** | **/** | **Y** | **/** | **Y** |  | **/** | **Y** | **/** | **/** | **/** | **Y** |  | **Y** | **/** | **Y** | **Y** | **Y** | **/** | **c.656G＞T**  **c.656G＞T** | **[17]** |
| **112** | **F** | **6** | **4** | **LD** | | **N** | **6** | **/** | **/** | **Y** | **/** | **Y** |  | **6** | **Y** | **/** | **/** | **/** | **Y** |  | **Y** | **/** | **Y** | **/** | **Y** | **/** | **c.656G＞T**  **c.656G＞T** | **[17]** |
| **113** | **F** | **7** | **7** | **Dysarthria** | | **Y** | **/** | **/** | **/** | **Y** | **/** | **N** |  | **/** | **Y** | **7** | **/** | **/** | **Y** | **7** | **Y** | **/** | **Y** | **/** | **Y** | **/** | **c.656G＞T**  **c.656G＞T** | **[17]** |
| **114** | **F** | **17** | **7** | **LLD** | | **N** | **17** | **N** | **16** | **Y** | **/** | **Y** |  | **7** | **Y** | **/** | **Y** | **/** | **Y** | **/** | **/** | **N** | **Y** | **Y** | **/** | **/** | **c.457A>T**  **c.519C>G** | **[65]** |
| **115** | **F** | **11** | **3** | **LLD** | | **Y** | **/** | **N** | **/** | **Y** | **/** | **Y** |  | **/** | **Y** | **/** | **Y** | **9** | **Y** | **9** | **/** | **/** | **Y** | **Y** | **Y** | **/** | **c.1145C＞T**  **c.1145C＞T** | **[18]** |
| **116** | **M** | **3.7** | **2.7** | **LLD** | | **Y** | **/** | **Y** | **/** | **Y** | **/** | **Y** |  | **2.7** | **N** | **/** | **N** | **/** | **N** | **/** | **/** | **Y** | **N** | **/** | **N** | **/** | **c.1561G＞A**  **c.1561G＞A** | **[12]** |
| **117** | **M** | **15** | **6** | **LLD** | | **Y** | **/** | **N** | **11** | **Y** | **/** | **Y** |  | **6** | **Y** | **/** | **N** | **/** | **Y** | **/** | **/** | **N** | **Y** | **Y** | **/** | **/** | **c.457A>T**  **c.519C>G** | **[65]** |
| **118** | **M** | **37** | **12** | **nyctalopia** | | **Y** | **/** | **N** | **/** | **Y** | **/** | **Y** |  | **/** | **Y** | **/** | **/** | **/** | **Y** | **/** | **/** | **/** | **/** | **N** | **Y** | **/** | **c.1010A＞C**  **c.1010A＞C** | **[41]** |
| **119** | **F** | **36** | **13** | **nyctalopia** | | **Y** | **/** | **N** | **/** | **Y** | **/** | **Y** |  | **/** | **Y** | **/** | **/** | **/** | **Y** | **/** | **/** | **/** | **/** | **/** | **Y** | **/** | **c.1010A＞C**  **c.1010A＞C** | **[41]** |
| **120** | **F** | **38** | **18** | **CD** | | **Y** | **/** | **Y** | **/** | **Y** | **/** | **Y** |  | **/** | **Y** | **/** | **/** | **/** | **Y** | **/** | **/** | **/** | **/** | **/** | **Y** | **/** | **c.1148A＞G**  **c.749delA** | **[41]** |
| **121** | **M** | **37** | **17** | **PS** | | **Y** | **/** | **Y** | **/** | **N** | **/** | **N** |  | **/** | **N** | **/** | **/** | **/** | **N** | **/** | **/** | **/** | **/** | **/** | **N** | **/** | **c.1397C＞T**  **c.1397C＞T** | **[41]** |
| **122** | **M** | **10** | **3** | **PBD** | | **Y** | **/** | **N** | **9** | **Y** | **/** | **Y** |  | **/** | **N** | **/** | **Y** | **/** | **Y** | **/** | **Y** | **/** | **Y** | **Y** | **Y** | **/** | **c.628+2T＞G**  **c.628+2T＞G** | **[10]** |
| **123** | **M** | **21** | **10** | **DD** | | **Y** | **/** | **Y** | **/** | **Y** | **16** | **Y** |  | **16** | **N** | **/** | **N** | **/** | **Y** | **/** | **/** | **Y** | **N** | **N** | **Y** | **/** | **c.1106C＞T**  **c.1106C＞T** | **[44]** |
| **124** | **F** | **34** | **24** | **IM** | | **Y** | **/** | **Y** | **/** | **N** | **/** | **N** |  | **/** | **N** | **/** | **N** | **/** | **Y** | **24** | **/** | **N** | **N** | **/** | **Y** | **/** | **c.115G＞T**  **c.803A＞G** | **[54]** |
| **125** | **F** | **41** | **15** | **LLD** | | **Y** | **/** | **Y** | **/** | **Y** | **37** | **Y** |  | **15** | **Y** | **/** | **Y** | **40** | **Y** | **37** | **/** | **N** | **N** | **/** | **Y** | **/** | **c.1130T＞C**  **IVS1-2A＞T** | **[47]** |
| **126** | **M** | **32** | **8** | **LLD** | | **Y** | **/** | **Y** | **/** | **N** | **/** | **Y** |  | **8** | **N** | **/** | **/** | **/** | **Y** | **26** | **/** | **N** | **N** | **N** | **Y** | **/** | **c.1130T＞C**  **IVS1-2A＞T** | **[47]** |
| **127** | **M** | **20** | **16** | **LLD** | | **N** | **20** | **N** | **/** | **Y** | **/** | **Y** |  | **16** | **Y** | **/** | **Y** | **/** | **Y** | **18** | **/** | **N** | **N** | **/** | **Y** | **/** | **c.1270C＞T**  **c.1270C＞T** | **[55]** |
| **128** | **M** | **27** | **17** | **/** | | **Y** | **/** | **Y** | **/** | **/** | **/** | **/** |  | **/** | **Y** | **/** | **N** | **/** | **Y** | **/** | **N** | **/** | **N** | **/** | **Y** | **/** | **c.803A＞G**  **c.1172T＞A** | **[55]** |
| **129** | **F** | **29** | **16** | **ULD** | | **Y** | **/** | **Y** | **/** | **Y** | **16** | **Y** |  | **16** | **Y** | **/** | **Y** | **16** | **Y** | **16** | **/** | **N** | **/** | **Y** | **Y** | **/** | **c.980T＞C**  **IVS4-1G＞T** | **[57]** |
| **130** | **F** | **57** | **28** | **ULD** | | **Y** | **/** | **Y** | **/** | **N** | **/** | **Y** |  | **/** | **N** | **/** | **Y** | **40** | **Y** | **40** | **N** | **N** | **/** | **/** | **Y** | **/** | **c.1037T＞G**  **c.1037T＞G** | **[32]** |
| **131** | **F** | **43** | **35** | **IM** | | **Y** | **/** | **Y** | **/** | **N** | **/** | **Y** |  | **/** | **N** | **/** | **N** | **/** | **N** | **/** | **N** | **N** | **/** | **/** | **Y** | **/** | **c.1037T＞G**  **c.1037T＞G** | **[32]** |
| **132** | **M** | **9** | **0.67** | **/** | | **N** | **9** | **/** | **/** | **/** | **/** | **Y** |  | **/** | **/** | **/** | **Y** | **/** | **N** | **/** | **N** | **/** | **/** | **Y** | **/** | **/** | **c.1231G＞A** | **[7]** |
| **133** | **M** | **17** | **10** | **/** | | **Y** | **/** | **/** | **/** | **Y** | **/** | **Y** |  | **/** | **/** | **/** | **N** | **/** | **Y** | **/** | **N** | **/** | **/** | **N** | **/** | **/** | **c.841A＞T**  **c.841A＞T** | **[7]** |
| **134** | **F** | **40** | **28** | **/** | | **Y** | **/** | **/** | **/** | **/** | **/** | **Y** |  | **/** | **/** | **/** | **N** | **/** | **Y** | **/** | **Y** | **/** | **/** | **N** | **/** | **/** | **c.370A＞G**  **c.1231G＞A** | **[7]** |
| **135** | **F** | **6** | **0.33** | **/** | | **Y** | **/** | **/** | **/** | **/** | **/** | **Y** |  | **/** | **/** | **/** | **Y** | **/** | **N** | **/** | **N** | **/** | **/** | **Y** | **/** | **/** | **c.943945del**  **c.1231G＞A** | **[7]** |
| **136** | **M** | **7** | **2** | **LLD** | | **Y** | **/** | **N** | **6** | **Y** | **4** | **Y** |  | **4** | **Y** | **4** | **/** | **/** | **Y** | **6** | **N** | **N** | **Y** | **/** | **Y** | **/** | **c.10171020delAGAT**  **c.10171020delAGAT** | **[38]** |
| **137** | **M** | **27** | **18** | **/** | | **Y** | **/** | **/** | **/** | **N** | **/** | **Y** |  | **/** | **/** | **/** | **N** | **/** | **Y** | **/** | **/** | **/** | **/** | **/** | **Y** | **/** | **c.464-465insC**  **c.1193C＞T** | **[19]** |
| **138** | **M** | **16** | **13** | **/** | | **Y** | **/** | **/** | **/** | **N** | **/** | **Y** |  | **/** | **/** | **/** | **N** | **/** | **Y** | **/** | **/** | **/** | **/** | **/** | **Y** | **/** | **c.398T＞A**  **c.970G＞T** | **[19]** |
| **139** | **M** | **16** | **10** | **/** | | **Y** | **/** | **/** | **/** | **Y** | **/** | **Y** |  | **/** | **/** | **/** | **N** | **/** | **Y** | **/** | **/** | **/** | **/** | **/** | **Y** | **/** | **c.745C＞T**  **c.970G＞T** | **[19]** |
| **140** | **M** | **46** | **24** | **/** | | **Y** | **/** | **/** | **/** | **Y** | **/** | **Y** |  | **/** | **/** | **/** | **N** | **/** | **Y** | **/** | **/** | **/** | **/** | **/** | **Y** | **/** | **c.1411A＞G**  **c.1510A＞G** | **[19]** |
| **141** | **M** | **40** | **30** | **/** | | **Y** | **/** | **/** | **/** | **N** | **/** | **N** |  | **/** | **N** | **/** | **N** | **/** | **Y** | **/** | **/** | **/** | **/** | **/** | **Y** | **/** | **c.970G＞T**  **c.1133A＞G** | **[19]** |
| **142** | **M** | **26** | **22** | **/** | | **Y** | **/** | **/** | **/** | **N** | **/** | **Y** |  | **/** | **/** | **/** | **N** | **/** | **Y** | **/** | **/** | **/** | **/** | **/** | **Y** | **/** | **c.1499A＞T**  **c.1133A＞G** | **[19]** |
| **143** | **M** | **45** | **25** | **LLD** | | **Y** | **/** | **Y** | **/** | **Y** | **/** | **Y** |  | **25** | **N** | **/** | **Y** | **35** | **Y** | **35** | **/** | **Y** | **Y** | **N** | **Y** | **/** | **c.445G＞T**  **c.1133A＞G** | **[43]** |
| **144** | **M** | **23** | **11** | **ULD** | | **Y** | **/** | **Y** | **/** | **N** | **/** | **Y** |  | **11** | **N** | **/** | **N** | **/** | **Y** | **15** | **Y** | **N** | **/** | **N** | **Y** | **/** | **c.1696C＞G**  **c.1160-1161insG** | **[58]** |
| **145** | **M** | **26** | **4** | **LLD** | | **Y** | **/** | **Y** | **/** | **Y** | **20** | **Y** |  | **20** | **Y** | **20** | **Y** | **20** | **Y** | **20** | **Y** | **Y** | **N** | **Y** | **Y** | **/** | **c.1561G＞A**  **c.1663G＞A** | **[15]** |
| **146** | **M** | **27** | **16** | **OMD** | | **Y** | **/** | **N** | **22** | **Y** | **16** | **Y** |  | **19** | **Y** | **/** | **Y** | **22** | **Y** | **27** | **/** | **N** | **/** | **/** | **Y** | **/** | **c.1583G＞A**  **c.1466T＞C** | **[59]** |
| **147** | **F** | **40** | **25** | **dysarthria** | | **Y** | **/** | **N** | **20** | **Y** | **17** | **Y** |  | **/** | **Y** | **18** | **Y** | **26** | **Y** | **15** | **N** | **/** | **/** | **/** | **/** | **/** | **c.1583C＞T**  **c.1583C＞T** | **[6]** |
| **148** | **M** | **32** | **18** | **dysarthria** | | **Y** | **/** | **N** | **21** | **Y** | **16** | **Y** |  | **/** | **Y** | **18** | **Y** | **19** | **Y** | **15** | **N** | **/** | **/** | **/** | **/** | **/** | **c.1583C＞T**  **c.1583C＞T** | **[6]** |
| **149** | **F** | **41** | **20** | **LD** | | **Y** | **/** | **N** | **25** | **Y** | **21** | **Y** |  | **20** | **Y** | **21** | **Y** | **20** | **Y** | **21** | **N** | **/** | **/** | **/** | **/** | **/** | **c.1583C＞T**  **c.1583C＞T** | **[6]** |
| **150** | **M** | **19** | **10** | **OMD** | | **Y** | **/** | **N** | **14** | **Y** | **10** | **Y** |  | **/** | **Y** | **14** | **Y** | **13** | **Y** | **13** | **Y** | **/** | **/** | **/** | **/** | **/** | **c.1583C＞T**  **c.1418_1424del** | **[6]** |
| **151** | **F** | **38** | **13** | **OMD** | | **Y** | **/** | **N** | **18** | **Y** | **13** | **Y** |  | **13** | **Y** | **18** | **Y** | **18** | **Y** | **16** | **Y** | **/** | **/** | **/** | **/** | **/** | **c.1583C＞T**  **c.1418_1424del** | **[6]** |
| **153** | **M** | **26** | **16** | **OMD** | | **Y** | **/** | **N** | **21** | **Y** | **16** | **Y** |  | **16** | **Y** | **21** | **Y** | **21** | **Y** | **19** | **N** | **/** | **/** | **/** | **/** | **/** | **c.1583C＞T**  **c.1418_1424del** | **[6]** |
| **154** | **M** | **58** | **18** | **PS** | | **Y** | **/** | **Y** | **/** | **Y** | **19** | **Y** |  | **18** | **N** | **/** | **Y** | **54** | **N** | **/** | **N** | **/** | **/** | **/** | **/** | **/** | **c.1583C＞T**  **c.1583C＞T** | **[6]** |
| **155** | **F** | **/** | **19** | **dysarthria** | | **Y** | **/** | **N** | **23** | **Y** | **21** | **Y** |  | **19** | **Y** | **23** | **Y** | **23** | **Y** | **20** | **N** | **/** | **/** | **/** | **/** | **/** | **c.1583C＞T**  **c.1418_1424del** | **[6]** |
| **152** | **F** | **23** | **7** | **LD** | | **Y** | **/** | **N** | **12** | **Y** | **10** | **Y** |  | **7** | **Y** | **10** | **Y** | **10** | **Y** | **10** | **Y** | **/** | **/** | **/** | **/** | **/** | **c.1583C＞T**  **c.1418_1424del** | **[6]** |
| **156** | **M** | **43** | **20** | **LLD** | | **Y** | **/** | **Y** | **/** | **/** | **/** | **Y** |  | **20** | **/** | **/** | **/** | **/** | **/** | **/** | **/** | **/** | **/** | **/** | **Y** | **/** | **c.1273-1275del**  **c.1233A＞Gl** | **[20]** |
| **157** | **F** | **19** | **18** | **PBD** | | **Y** | **/** | **Y** | **/** | **/** | **/** | **Y** |  | **19** | **/** | **/** | **/** | **/** | **/** | **/** | **Y** | **/** | **/** | **/** | **Y** | **/** | **p.R286C***  **p.R286C*** | **[48]** |
| **158** | **F** | **49** | **47** | **LLD** | | **Y** | **/** | **Y** | **/** | **N** | **/** | **Y** |  | **47** | **N** | **/** | **/** | **/** | **/** | **/** | **N** | **N** | **/** | **/** | **Y** | **/** | **c.1133A＞G**  **c.1319G＞C** | **[25]** |
| **159** | **M** | **43** | **19** | **ULD** | | **Y** | **/** | **Y** | **/** | **N** | **/** | **Y** |  | **19** | **N** | **/** | **Y** | **/** | **Y** | **/** | **N** | **/** | **N** | **/** | **Y** | **/** | **c.1133A＞G**  **c.1273_1275del** | **[23]** |
| **160** | **M** | **34** | **23** | **OMD** | | **Y** | **/** | **Y** | **/** | **Y** | **23** | **Y** |  | **/** | **N** | **/** | **Y** | **/** | **Y** | **/** | **N** | **/** | **N** | **/** | **Y** | **/** | **c.1133A＞G**  **c.1500_1501del** | **[23]** |
| **161** | **M** | **62** | **48** | **PS** | | **Y** | **/** | **Y** | **/** | **N** | **/** | **Y** |  | **/** | **N** | **/** | **N** | **/** | **N** | **/** | **N** | **/** | **N** | **/** | **Y** | **/** | **c.1153delCinsTT**  **c.1319G＞C** | **[23]** |
| **162** | **M** | **59** | **25** | **ULD** | | **Y** | **/** | **N** | **/** | **N** | **/** | **Y** |  | **25** | **Y** | **/** | **Y** | **/** | **Y** | **/** | **N** | **/** | **N** | **/** | **Y** | **/** | **c.1153delCinsTT**  **c.1319G＞C** | **[23]** |
| **163** | **M** | **40** | **35** | **LLD** | | **Y** | **/** | **N** | **/** | **N** | **/** | **Y** |  | **35** | **N** | **/** | **N** | **/** | **N** | **/** | **N** | **/** | **Y** | **/** | **Y** | **/** | **c.1133A＞G**  **c.1319G＞C** | **[23]** |
| **164** | **F** | **50** | **24** | **PS** | | **Y** | **/** | **Y** | **/** | **N** | **/** | **Y** |  | **/** | **N** | **/** | **N** | **/** | **Y** | **24** | **N** | **/** | **N** | **/** | **Y** | **/** | **c.1133A＞G**  **c.1319G＞C** | **[23]** |
| **165** | **M** | **22** | **15** | **ULD** | | **Y** | **/** | **Y** | **/** | **Y** | **/** | **Y** |  | **15** | **Y** | **/** | **Y** | **/** | **Y** | **/** | **Y** | **/** | **N** | **/** | **Y** | **/** | **c.1003A > G,**  **c.1270_1272del** | **[23]** |
| **166** | **M** | **20** | **15** | **LLD** | | **Y** | **/** | **Y** | **/** | **N** | **/** | **Y** |  | **15** | **N** | **/** | **N** | **/** | **Y** | **/** | **N** | **/** | **N** | **/** | **Y** | **/** | **c.1003A > G,**  **c.1270_1272del** | **[23]** |
| **167** | **M** | **43** | **42** | **LLD** | | **Y** | **/** | **Y** | **/** | **N** | **/** | **Y** |  | **42** | **N** | **/** | **N** | **/** | **N** | **/** | **N** | **/** | **N** | **/** | **Y** | **/** | **c.1133A＞G**  **c.1273_1275del** | **[23]** |
| **168** | **M** | **33** | **21** | **ULD** | | **Y** | **/** | **Y** | **/** | **N** | **/** | **Y** |  | **21** | **N** | **/** | **N** | **/** | **N** | **/** | **N** | **/** | **N** | **/** | **Y** | **/** | **c.1003A＞G**  **c.1003A＞G** | **[23]** |
| **169** | **M** | **45** | **35** | **PS** | | **Y** | **/** | **Y** | **/** | **N** | **/** | **N** |  | **/** | **N** | **/** | **N** | **/** | **N** | **/** | **N** | **/** | **N** | **/** | **Y** | **/** | **c.1133A＞G**  **c.1154_1155insTT** | **[23]** |
| **170** | **M** | **65** | **38** | **ULD** | | **Y** | **/** | **N** | **/** | **N** | **/** | **Y** |  | **38** | **Y** | **/** | **N** | **/** | **Y** | **/** | **N** | **/** | **N** | **/** | **Y** | **/** | **c.1133A＞G**  **c.823_824del** | **[23]** |
| **171** | **M** | **62** | **52** | **PS** | | **Y** | **/** | **Y** | **/** | **N** | **/** | **N** |  | **/** | **N** | **/** | **N** | **/** | **Y** | **/** | **N** | **/** | **N** | **/** | **Y** | **/** | **c.1133A＞G**  **c.1319G＞C** | **[23]** |
| **172** | **M** | **47** | **28** | **ULD** | | **Y** | **/** | **N** | **/** | **N** | **/** | **Y** |  | **28** | **N** | **/** | **N** | **/** | **Y** | **/** | **N** | **/** | **N** | **/** | **Y** | **/** | **c.1133A＞G**  **c.1319G＞C** | **[23]** |
| **173** | **F** | **49** | **47** | **LLD** | | **Y** | **/** | **Y** | **/** | **N** | **/** | **Y** |  | **47** | **N** | **/** | **N** | **/** | **N** | **/** | **Y** | **/** | **N** | **/** | **Y** | **/** | **c.1133A＞G**  **c.1319G＞C** | **[23]** |
| **174** | **M** | **/** | **30** | **ULD** | | **Y** | **/** | **Y** | **/** | **N** | **/** | **Y** |  | **30** | **N** | **/** | **N** | **/** | **Y** | **/** | **N** | **/** | **/** | **/** | **Y** | **/** | **p.N294I***  **p.N294I*** | **[8]** |
| **175** | **F** | **/** | **16** | **dysarthria** | | **/** | **/** | **/** | **/** | **/** | **/** | **Y** |  | **16** | **N** | **/** | **/** | **/** | **N** | **/** | **N** | **/** | **/** | **/** | **Y** | **/** | **p.N294I***  **p.N294I*** | **[8]** |
| **176** | **M** | **/** | **26** | **PS** | | **/** | **/** | **/** | **/** | **N** | **/** | **N** |  | **/** | **N** | **/** | **N** | **/** | **Y** | **/** | **N** | **/** | **Y** | **/** | **Y** | **/** | **p.N294I***  **p.N294I*** | **[8]** |
| **177** | **M** | **25** | **21** | **LD** | | **Y** | **/** | **Y** | **/** | **N** | **/** | **Y** |  | **21** | **N** | **/** | **N** | **/** | **N** | **/** | **N** | **N** | **N** | **N** | **Y** | **/** | **c.673A＞G**  **c.673A＞G** | **[35]** |
| **178** | **M** | **26** | **25** | **dysarthria** | | **Y** | **/** | **Y** | **/** | **Y** | **25** | **N** |  | **/** | **N** | **/** | **N** | **/** | **Y** | **25** | **N** | **N** | **N** | **/** | **Y** | **/** | **c.260A>G**  **c.405dupC** | **[36]** |
| **179** | **F** | **38** | **12** | **PS** | | **Y** | **/** | **Y** | **/** | **N** | **/** | **N** |  | **/** | **N** | **/** | **N** | **/** | **Y** | **12** | **N** | **N** | **N** | **N** | **Y** | **/** | **c.1208A>T**  **c.1208A>T** | **[13]** |
| **180** | **M** | **30** | **24** | **PS** | | **Y** | **/** | **Y** | **/** | **N** | **/** | **Y** |  | **/** | **N** | **/** | **N** | **/** | **Y** | **/** | **N** | **N** | **N** | **/** | **Y** | **/** | **c.833G＞T**  **c.833G＞T** | **[13]** |
| **181** | **M** | **25** | **22** | **PS** | | **Y** | **/** | **Y** | **/** | **N** | **/** | **N** |  | **/** | **N** | **/** | **N** | **/** | **N** | **/** | **N** | **N** | **Y** | **/** | **Y** | **/** | **c.1583C＞T**  **c.1583C＞T** | **[13]** |
| **182** | **M** | **40** | **35** | **IM** | | **Y** | **/** | **Y** | **/** | **N** | **/** | **Y** |  | **/** | **N** | **/** | **N** | **/** | **N** | **/** | **N** | **N** | **/** | **N** | **Y** | **/** | **c.1133A＞G**  **c.1319A＞C** | **[52]** |
| **183** | **M** | **41** | **6** | **nyctalopia** | | **Y** | **/** | **Y** | **/** | **Y** | **/** | **Y** |  | **/** | **Y** | **/** | **N** | **/** | **Y** | **15** | **Y** | **N** | **N** | **Y** | **Y** | **/** | **c.71A＞G**  **c.71A＞G** | **[37]** |
| **184** | **M** | **33** | **22** | **LLD** | | **Y** | **/** | **N** | **33** | **N** | **/** | **Y** |  | **22** | **Y** | **33** | **Y** | **33** | **Y** | **27** | **Y** | **N** | **N** | **N** | **Y** | **/** | **c.695A＞G**  **c.695A＞G** | **[9]** |
| **185** | **M** | **8** | **/** | **LLD** | | **Y** | **/** | **N** | **/** | **/** | **/** | **Y** |  | **/** | **Y** | **/** | **/** | **/** | **Y** | **/** | **N** | **Y** | **N** | **Y** | **Y** | **/** | **p.L385CfsX13***  **p.R440P*** | **[31]** |
| **186** | **F** | **25** | **2** | **LLD** | | **Y** | **/** | **N** | **25** | **N** | **/** | **Y** |  | **2** | **N** | **/** | **Y** | **4** | **Y** | **13** | **N** | **Y** | **Y** | **N** | **Y** | **/** | **c.1069C＞T**  **c.1069C＞T** | **[39]** |
| **187** | **F** | **26** | **7** | **LLD** | | **Y** | **/** | **N** | **14** | **Y** | **/** | **Y** |  | **7** | **Y** | **15** | **Y** | **/** | **Y** | **/** | **Y** | **N** | **Y** | **Y** | **Y** | **/** | **c.457A>T**  **c.519C>G** | **[65]** |
| **188** | **F** | **7** | **1** | **LLD** | | **Y** | **/** | **N** | **7** | **Y** | **/** | **Y** |  | **1** | **Y** | **/** | **/** | **/** | **Y** | **/** | **N** | **N** | **Y** | **/** | **Y** | **/** | **c.982-1G>C**  **c.982-1G>C** | **[1]** |
| **189** | **F** | **9** | **9** | **LLD** | | **Y** | **/** | **Y** | **/** | **N** | **/** | **Y** |  | **9** | **N** | **/** | **N** | **/** | **N** | **/** | **Y** | **N** | **Y** | **/** | **N** | **/** | **c.970G＞T**  **c.1133A＞G** | **[45]** |
| **190** | **F** | **10** | **8** | **LD** | | **Y** | **/** | **Y** | **/** | **N** | **/** | **Y** |  | **8** | **N** | **/** | **N** | **/** | **N** | **/** | **N** | **N** | **N** | **/** | **Y** | **/** | **c.808C＞G**  **c.1103A＞G** | **[45]** |
| **191** | **M** | **9** | **1** | **LLD** | | **Y** | **/** | **N** | **9** | **Y** | **/** | **Y** |  | **1** | **Y** | **/** | **/** | **/** | **Y** | **/** | **N** | **N** | **Y** | **Y** | **Y** | **/** | **c.982-1G>C**  **c.982-1G>C** | **[1]** |
| **192** | **M** | **4** | **2** | **LLD** | | **N** | **4** | **N** | **3** | **Y** | **/** | **Y** |  | **2** | **Y** | **/** | **/** | **/** | **Y** | **/** | **N** | **N** | **Y** | **N** | **N** | **/** | **c.982-1G>C**  **c.982-1G>C** | **[1]** |
| **193** | **M** | **53** | **25** | **PS** | | **Y** | **/** | **Y** | **/** | **N** | **/** | **Y** |  | **/** | **N** | **/** | **N** | **/** | **Y** | **50** | **N** | **N** | **Y** | **N** | **Y** | **/** | **c.1561G＞A**  **c.1585A＞G** | **[21]** |
| **194** | **M** | **4** | **1** | **DD** | | **N** | **4** | **N** | **3** | **Y** | **/** | **Y** |  | **1** | **Y** | **/** | **/** | **/** | **N** | **/** | **N** | **N** | **Y** | **/** | **N** | **/** | **c.809T>C**  **c.809T>C** | **[1]** |
| **195** | **F** | **41** | **15** | **LLD** | | **Y** | **/** | **Y** | **/** | **/** | **/** | **Y** |  | **15** | **N** | **/** | **Y** | **/** | **Y** | **/** | **N** | **N** | **/** | **N** | **Y** | **/** | **IVS1-2＞T**  **c.258T＞C** | **[46]** |
| **196** | **M** | **32** | **8** | **PS** | | **Y** | **/** | **Y** | **/** | **/** | **/** | **Y** |  | **/** | **N** | **/** | **N** | **/** | **Y** | **/** | **N** | **N** | **/** | **N** | **Y** | **/** | **IVS1-2＞T**  **c.258T＞C** | **[46]** |
| **197** | **M** | **11** | **1** | **CD** | | **Y** | **/** | **Y** | **/** | **Y** | **/** | **Y** |  | **/** | **Y** | **/** | **/** | **/** | **Y** | **/** | **N** | **N** | **N** | **N** | **Y** | **/** | **c.982-1G>C**  **c.1583C>T** | **[1]** |
| **198** | **M** | **15** | **5** | **LLD** | | **N** | **15** | **N** | **12** | **N** | **/** | **Y** |  | **5** | **N** | **/** | **N** | **/** | **Y** | **11** | **Y** | **Y** | **N** | **/** | **Y** | **/** | **c.1021T>G**  **c.1687C>G** | **[51]** |
| **199** | **F** | **11** | **2** | **LLD** | | **Y** | **/** | **Y** | **/** | **/** | **/** | **Y** |  | **2** | **N** | **/** | **N** | **/** | **Y** | **/** | **N** | **Y** | **Y** | **/** | **N** | **/** | **c.1555T>C**  **c.1555T>C** | **[51]** |
| **200** | **F** | **7** | **6** | **LLD** | | **Y** | **/** | **Y** | **/** | **/** | **/** | **Y** |  | **6** | **N** | **/** | **Y** | **/** | **Y** | **/** | **N** | **Y** | **N** | **/** | **Y** | **/** | **c.1555T>C**  **c.1469C>G** | **[51]** |
| **201** | **F** | **16** | **3** | **ULD** | | **Y** | **/** | **Y** | **/** | **/** | **/** | **Y** |  | **3** | **N** | **/** | **Y** | **/** | **Y** | **/** | **N** | **Y** | **N** | **/** | **N** | **/** | **c.1021T>G**  **c.1355A>G** | **[51]** |
| **202** | **M** | **15** | **9** | **LLD** | | **Y** | **/** | **Y** | **/** | **/** | **/** | **Y** |  | **9** | **/** | **/** | **Y** | **/** | **Y** | **/** | **N** | **Y** | **Y** | **/** | **N** | **/** | **c.784G>A**  **c.1572G>C** | **[51]** |
| **203** | **M** | **15** | **2** | **LLD** | | **Y** | **/** | **N** | **14** | **/** | **/** | **Y** |  | **2** | **/** | **/** | **Y** | **/** | **Y** | **/** | **N** | **N** | **Y** | **Y** | **N** | **/** | **c.856C>T**  **c.644G>A** | **[51]** |
| **204** | **F** | **13** | **2** | **LLD** | | **N** | **13** | **N** | **7** | **/** | **/** | **Y** |  | **2** | **/** | **/** | **Y** | **/** | **Y** | **/** | **N** | **Y** | **Y** | **N** | **N** | **Y** | **c.1325A>G c.1325A>G** | **[51]** |
| **205** | **F** | **6** | **4** | **LLD** | | **Y** | **/** | **Y** | **/** | **/** | **/** | **Y** |  | **4** | **/** | **/** | **Y** | **/** | **Y** | **/** | **N** | **Y** | **N** | **/** | **N** | **/** | **c.475DelC**  **c.475DelC** | **[51]** |
| **206** | **M** | **6** | **2** | **LLD** | | **N** | **6** | **Y** | **/** | **/** | **/** | **Y** |  | **2** | **/** | **/** | **Y** | **/** | **Y** | **/** | **N** | **N** | **N** | **N** | **Y** | **/** | **c.1502T＞C**  **c.1502T＞C** | **[51]** |
| **207** | **F** | **18** | **2** | **LLD** | | **N** | **18** | **N** | **11** | **/** | **/** | **Y** |  | **2** | **/** | **/** | **N** | **/** | **N** | **/** | **N** | **N** | **N** | **Y** | **Y** | **Y** | **c.1319G>C**  **c.1630G>T** | **[51]** |
| **208** | **F** | **13** | **3** | **LLD** | | **Y** | **/** | **N** | **13** | **/** | **/** | **Y** |  | **3** | **/** | **/** | **N** | **/** | **Y** | **/** | **N** | **N** | **N** | **/** | **Y** | **/** | **c.970G>T**  **c.1502T＞C** | **[51]** |
| **209** | **F** | **20** | **8** | **LLD** | | **Y** | **/** | **N** | **13** | **Y** | **/** | **Y** |  | **8** | **Y** | **/** | **Y** | **/** | **Y** | **/** | **N** | **N** | **Y** | **/** | **N** | **/** | **c.1469C>T**  **c.1502T＞C** | **[51]** |
| **210** | **F** | **22** | **11** | **EMD** | | **Y** | **/** | **N** | **18** | **Y** | **/** | **Y** |  | **/** | **Y** | **/** | **Y** | **16** | **Y** | **20** | **N** | **N** | **N** | **/** | **Y** | **/** | **c.721G>A**  **c.721G>A** | **[51]** |
| **211** | **F** | **11** | **6** | **LLD** | | **Y** | **/** | **Y** | **/** | **/** | **/** | **Y** |  | **6** | **Y** | **8** | **Y** | **/** | **Y** | **/** | **N** | **N** | **Y** | **Y** | **Y** | **/** | **c.1171_1174dupAT**  **c.519C>G** | **[51]** |
| **212** | **F** | **14** | **9** | **ULD** | | **Y** | **/** | **Y** | **/** | **/** | **/** | **Y** |  | **9** | **/** | **/** | **Y** | **/** | **Y** | **/** | **N** | **Y** | **N** | **Y** | **N** | **/** | **c.445G>T**  **c.1270C>T** | **[51]** |
| **213** | **F** | **8** | **1** | **DD** | | **Y** | **/** | **N** | **7** | **/** | **/** | **Y** |  | **/** | **Y** | **8** | **Y** | **6** | **Y** | **6** | **N** | **Y** | **N** | **/** | **Y** | **/** | **c.108+3A>G**  **c.108+3A>G** | **[51]** |
| **214** | **M** | **12** | **1** | **LLD** | | **Y** | **/** | **N** | **10** | **Y** | **9** | **Y** |  | **1** | **Y** | **10** | **N** | **/** | **Y** | **1** | **N** | **Y** | **N** | **/** | **Y** | **Y** | **c.1355A＞G**  **c.1555T＞C** | **[51]** |
| **215** | **F** | **11** | **6** | **LLD** | | **Y** | **/** | **Y** | **/** | **Y** | **10** | **Y** |  | **6** | **/** | **/** | **Y** | **11** | **/** | **/** | **N** | **Y** | **/** | **Y** | **Y** | **/** | **c.67A>G**  **c.67A>G** | **[51]** |
| **216** | **F** | **9** | **1** | **nyctalopia** | | **Y** | **/** | **Y** | **/** | **Y** | **8** | **Y** |  | **6** | **N** | **/** | **N** | **/** | **1** | **1** | **N** | **Y** | **N** | **Y** | **Y** | **/** | **c.1324G＞T**  **c.1324G＞T** | **[51]** |
| **217** | **M** | **10** | **2** | **LLD** | | **Y** | **/** | **N** | **6** | **Y** | **7** | **Y** |  | **2** | **Y** | **7** | **Y** | **5** | **Y** | **4** | **N** | **N** | **N** | **/** | **Y** | **/** | **c.650A＞G**  **c.468T＞G** | **Our**  **Center** |
| **218** | **F** | **8** | **7** | **ULD** | | **Y** | **/** | **Y** | **/** | **N** | **/** | **Y** |  | **7** | **N** | **/** | **N** | **/** | **N** | **/** | **N** | **N** | **N** | **N** | **Y** | **Y** | **c.856C＞T**  **c.1502T＞A** | **Our**  **Center** |
| **219** | **M** | **11** | **8** | **ULD** | | **Y** | **/** | **Y** | **/** | **N** | **/** | **Y** |  | **8** | **N** | **/** | **N** | **/** | **N** | **/** | **N** | **N** | **N** | **N** | **Y** | **/** | **c.97G＞T**  **c.97G＞T** | **Our**  **Center** |
| **220** | **M** | **12** | **2** | **LLD** | | **Y** | **/** | **N** | **12** | **/** | **/** | **Y** |  | **2** | **Y** | **12** | **N** | **/** | **N** | **/** | **N** | **N** | **N** | **Y** | **N** | **/** | **c.989G＞C**  **c.1300G＞T** | **Our**  **Center** |
| **221** | **M** | **5** | **2** | **LLD** | | **Y** | **/** | **N** | **5** | **/** | **/** | **Y** |  | **2** | **Y** | **5** | **/** | **/** | **Y** | **5** | **N** | **N** | **N** | **/** | **Y** | **/** | **c.1172T＞A**  **c.1172T＞A** | **Our**  **Center** |
| **222** | **M** | **12** | **4** | **LLD** | | **Y** | **/** | **N** | **12** | **/** | **/** | **Y** |  | **4** | **/** | **/** | **/** | **/** | **/** | **./** | **N** | **N** | **N** | **/** | **Y** | **/** | **c.611C＞G**  **c.274G＞A** | **Our**  **Center** |
| **223** | **M** | **6** | **4** | **LD** | | **Y** | **/** | **Y** | **/** | **/** | **/** | **Y** |  | **4** | **/** | **/** | **/** | **/** | **/** | **./** | **/** | **Y** | **N** | **/** | **N** | **/** | **c.145DelC**  **c.145DelC** | **[62]** |
| **224** | **F** | **8** | **6** | **LLD** | | **Y** | **/** | **N** | **7** | **/** | **/** | **Y** |  | **6** | **/** | **/** | **/** | **/** | **/** | **./** | **/** | **N** | **Y** | **/** | **N** | **Y** | **c.995A＞G** | **[62]** |
| **225** | **M** | **15** | **2** | **LLD** | | **Y** | **/** | **N** | **15** | **/** | **/** | **Y** |  | **2** | **/** | **/** | **Y** | **13** | **Y** | **13** | **N** | **N** | **N** | **Y** | **N** | **/** | **c.526C＞T**  **c.314G＞A** | **[62]** |
| **226** | **M** | **16** | **9** | **LLD** | | **Y** | **/** | **N** | **16** | **/** | **/** | **Y** |  | **9** | **/** | **/** | **/** | **/** | **Y** | **13** | **N** | **N** | **N** | **/** | **N** | **/** | **c.1242G＞C** | **[62]** |
| **227** | **F** | **10** | **8** | **ULD** | | **Y** | **/** | **N** | **10** | **/** | **/** | **Y** |  | **8** | **/** | **/** | **/** | **/** | **/** | **/** | **/** | **N** | **N** | **/** | **Y** | **/** | **c.691T＞G**  **c.1025A＞G** | **[62]** |
| **228** | **F** | **6** | **1** | **LLD** | | **/** | **/** | **N** | **6** | **/** | **/** | **Y** |  | **1** | **/** | **/** | **/** | **/** | **/** | **/** | **/** | **N** | **Y** | **N** | **N** | **/** | **c.460C＞T** | **[62]** |
| **229** | **F** | **14** | **9** | **ULD** | | **Y** | **/** | **N** | **11** | **Y** | **14** | **Y** |  | **9** | **/** | **/** | **/** | **/** | **Y** | **9** | **Y** | **Y** | **N** | **Y** | **Y** | **/** | **c.115G＞T**  **c.940C＞T** | **[62]** |
| **230** | **M** | **10** | **7** | **ULD** | | **Y** | **/** | **Y** | **/** | **Y** | **9** | **Y** |  | **7** | **Y** | **9** | **Y** | **8** | **Y** | **8** | **N** | **N** | **N** | **N** | **Y** | **N** | **c.1502T＞A**  **c.833G＞A** | **[62]** |
| **231** | **F** | **14** | **2** | **LLD** | | **Y** | **/** | **Y** | **/** | **N** | **/** | **Y** |  | **2** | **N** | **/** | **N** | **/** | **N** | **/** | **N** | **N** | **Y** | **N** | **Y** | **N** | **c.53G＞A** | **[62]** |
| **232** | **F** | **16** | **11** | **CD** | | **Y** | **/** | **N** | **16** | **Y** | **15** | **Y** |  | **15** | **/** | **/** | **/** | **/** | **Y** | **15** | **N** | **N** | **Y** | **/** | **Y** | **/** | **c.391G＞A** | **[62]** |
| **233** | **F** | **13** | **8** | **LLD** | | **Y** | **/** | **N** | **13** | **/** | **/** | **Y** |  | **8** | **/** | **/** | **/** | **/** | **/** | **/** | **/** | **N** | **N** | **/** | **N** | **/** | **c.1139C＞T**  **c.1172T＞C** | **[62]** |
| **234** | **F** | **13** | **3** | **LLD** | | **Y** | **/** | **N** | **13** | **/** | **/** | **Y** |  | **3** | **/** | **/** | **/** | **/** | **/** | **/** | **/** | **N** | **/** | **/** | **Y** | **/** | **c.640G＞T**  **c.1172T＞A** | **[62]** |
| **235** | **M** | **21** | **20** | **ULD** | | **Y** | **/** | **Y** | **/** | **Y** | **21** | **Y** |  | **20** | **N** | **/** | **/** | **/** | **Y** | **21** | **Y** | **N** | **N** | **Y** | **Y** | **/** | **c. 856C＞T**  **c. 856C＞T** | **[63]** |
| **236** | **M** | **20** | **10** | **LLD** | | **Y** | **/** | **Y** | **/** | **Y** | **20** | **Y** |  | **10** | **N** | **/** | **N** | **/** | **Y** | **18** | **Y** | **N** | **N** | **N** | **Y** | **/** | **c. 856C＞T**  **c. 856C＞T** | **[63]** |
| **237** | **M** | **54** | **30** | **PS** | | **Y** | **/** | **Y** | **/** | **N** | **/** | **Y** |  | **37** | **N** | **/** | **N** | **/** | **Y** | **54** | **Y** | **N** | **Y** | **/** | **Y** | **/** | **c. 775G＞A**  **c. 1441C＞T** | **[64]** |
| **238** | **F** | **32** | **16** | **LLD** | | **Y** | **/** | **Y** | **/** | **/** | **/** | **Y** |  | **16** | **N** | **/** | **/** | **/** | **/** | **/** | **/** | **N** | **/** | **N** | **Y** | **/** | **c.706_708delGAA**  **c.706_708delGAA** | **[66]** |
| **239** | **M** | **30** | **18** | **PS** | | **Y** | **/** | **Y** | **/** | **/** | **/** | **Y** |  | **/** | **Y** | **18** | **/** | **/** | **/** | **/** | **/** | **N** | **/** | **N** | **Y** | **/** | **c.706_708delGAA**  **c.706_708delGAA** | **[66]** |
| **240** | **M** | **13** | **13** | **tic** | | **Y** | **/** | **Y** | **/** | **N** | **/** | **Y** |  | **13** | **N** | **/** | **N** | **/** | **N** | **/** | **Y** | **N** | **N** | **/** | **Y** | **/** | **c. 833G＞T**  **c. 833G＞T** | **[42]** |
| **241** | **M** | **55** | **48** | **PS** | | **Y** | **/** | **Y** | **/** | **N** | **/** | **N** |  | **/** | **N** | **/** | **N** | **/** | **N** | **/** | **N** | **N** | **N** | **/** | **Y** | **/** | **c.1153delCinsTT**  **c.1319G>C** | **[27]** |
| **242** | **M** | **25** | **14** | **ULD** | | **Y** | **/** | **Y** | **/** | **Y** | **19** | **Y** |  | **14** | **Y** | **19** | **/** | **/** | **Y** | **/** | **/** | **N** | **/** | **/** | **N** | **/** | **c.332T＞A**  **c.332T＞A** | **[14]** |
| **243** | **M** | **25** | **22** | **ULD** | | **Y** | **/** | **Y** | **/** | **Y** | **25** | **Y** |  | **22** | **N** | **/** | **N** | **/** | **Y** | **25** | **N** | **N** | **N** | **N** | **Y** | **/** | **c.1424T＞C**  **c.1583C>T** | **[4]** |
| **244** | **M** | **14** | **13** | **LLD** | | **Y** | **/** | **Y** | **/** | **Y** | **14** | **Y** |  | **13** | **N** | **/** | **N** | **/** | **Y** | **13** | **Y** | **N** | **/** | **Y** | **Y** | **/** | **c.1217A>T**  **c.1217A>T** | **[3]** |
| **245** | **F** | **17** | **11** | **ULD** | | **Y** | **/** | **N** | **17** | **Y** | **17** | **Y** |  | **11** | **Y** | **12** | **Y** | **17** | **/** | **/** | **N** | **N** | **N** | **N** | **Y** | **/** | **c.628 + 2T > G**  **c.628 + 2T > G** | **[2]** |
| **246** | **M** | **12** | **3** | **LLD** | | **N** | **12** | **N** | **10** | **Y** | **/** | **Y** |  | **3** | **Y** | **10** | **Y** | **/** | **Y** | **/** | **/** | **N** | **Y** | **Y** | **/** | **/** | **c.846_847delAG**  **c.846_847delAG** | **[65]** |
| **247** | **F** | **13** | **3** | **LLD** | | **N** | **13** | **N** | **11** | **Y** | **/** | **Y** |  | **3** | **Y** | **10** | **Y** | **/** | **Y** | **/** | **/** | **N** | **Y** | **Y** | **/** | **/** | **c.846_847delAG**  **c.846_847delAG** | **[65]** |
| **248** | **F** | **22** | **5** | **LLD** | | **Y** | **/** | **N** | **10** | **Y** | **/** | **Y** |  | **5** | **Y** | **15** | **Y** | **/** | **Y** | **/** | **/** | **N** | **Y** | **Y** | **Y** | **/** | **c.1171_1174dupAT c.1171_1174dupAT** | **[65]** |

S:sex ,M:male, F:female, AALFW:age at last follow up ,AAO:age at onset,IS: Initial symptom,LOA: loss of independent ambulance,OMD:oromandibular dystonia,LD:limb dystonia,LLD:lower limb dystonia,ULD:upper limb dystonia,GD:general dystonia,PBD: Psychological and behavior disturbance,DD: Developmental delay,GPC:calcification globus pallidus,EOTS;Eye Of tiger sign,CD:cervical dystonia,IM: involuntary movement,VL:vision loss,PS: Parkinson sign,Ref: reference,Y:Yes,N:No

*is protein change not *PANK2* mutation,because the mutation is not identified in the article,so this is protein change

“/”means not mentioned in the reported studies

**References**

1. Sakpichaisakul K, Saengow VE, Suwanpratheep P, Rongnoparat K, Panthan B, Trachoo O. Novel PANK2 mutation discovered among South East Asian children living in Thailand affected with pantothenate kinase associated neurodegeneration. *J Clin Neurosci.* 2019;66:187-190.

2. Li H, Zhang C, Pan Y, Sun B, Li D, Wu Y. Status dystonicus in pantothenate kinase-associated neurodegeneration due to internal pulse generator depletion: Case study and literature review. *J Neurol Sci.* 2019;400:44-46

3. Israni A, Mandal A. Atypical pantothenate kinase-associated neurodegeneration with novel genetic mutation. *Neurology India.* 2017;65(4):914-915.

4. Paraskevas GP, Yapijakis C, Bougea A, et al. Novel PANK2 mutation in the first Greek compound heterozygote patient with pantothenate-kinase-associated neurodegeneration. *SAGE open medical case reports.* 2017;5:2050313X17720101.

5. Valentino P, Annesi G, Cirò Candiano IC, et al. Genetic heterogeneity in patients with pantothenate kinase–associated neurodegeneration and classic magnetic resonance imaging eye‐of‐the‐tiger pattern. Mov Disord. 2006;21(2):252-254

6. Tomić A, Petrović I, Svetel M, Dobričić V, Mišković ND, Kostić VS. Pattern of disease progression in atypical form of pantothenate-kinase-associated neurodegeneration (PKAN)–Prospective study. *Parkinsonism Relat Disord.* 2015;21(5):521-524.

7. Thomas M, Hayflick SJ, Jankovic J. Clinical heterogeneity of neurodegeneration with brain iron accumulation (Hallervorden‐Spatz syndrome) and pantothenate kinase‐associated neurodegeneration. *Mov Disord.* 2004;19(1):36-42.

8. Camargos S, Gurgel-Giannetti J, Lees A, Hardy J, Singleton A, Cardoso F. Low prevalence of PANK2 mutations in Brazilian patients with early onset generalised dystonia and basal ganglia abnormalities on MRI. *Journal of Neurology, Neurosurgery Psychiatry.* 2011;82(9):1059-1060.

9. Tanteles GA, Spanou-Aristidou E, Antoniou C, Christophidou-Anastasiadou V, Kleopa KA. Novel homozygous PANK2 mutation causing atypical pantothenate kinase-associated neurodegeneration (PKAN) in a Cypriot family. *J Neurol Sci.* 2014;340(1-2):233-236.

10. Tanrıkulu B, Özen A, Günal DI, et al. Deep brain stimulation as treatment for dystonic storm in pantothenate kinase-associated neurodegeneration syndrome: case report of a patient with homozygous C. 628 2 T> G mutation of the PANK2 gene. Acta Neurochir (Wien). 2015;157(9):1513-1517.

11. Shi X, Zheng F, Ye X, et al. Basal ganglia calcification and novel compound heterozygous mutations in the PANK2 gene in a Chinese boy with classic pantothenate kinase-associated neurodegeneration: a case report. Medicine (Baltimore). 2018;97(15).

12. Rossi D, De Grandis E, Barzaghi C, et al. Early-onset neurodegeneration with brain iron accumulation due to PANK2 mutation. Brain Dev. 2012;34(6):536-538.

13. Rohani M, Shahidi G, Alavi A, et al. Tremor‐Dominant Pantothenate Kinase‐associated Neurodegeneration. Mov Disord Clin Pract. 2017;4(5):772-774.

14. Pratini NR, Sweeters N, Vichinsky E, Neufeld JA. Treatment of Classic Pantothenate Kinase-Associated Neurodegeneration (PKAN) with Deferiprone and Intrathecal Baclofen. Am J Phys Med Rehabil. 2013;92(8):728-733.

15. Pérez-González E, Chacón-Camacho O, Arteaga-Vázquez J, Zenteno J, Mutchinick，OM. A novel gene mutation in PANK2 in a patient with an atypical form of pantothenate kinase-associated neurodegeneration. Eur J Med Genet. 2013;56(11):606-608.

16. Pellecchia MT, Valente E, Cif L, et al. The diverse phenotype and genotype of pantothenate kinase-associated neurodegeneration. Neurology. 2005;64(10):1810-1812. 17. Morales‐Briceño H, Chacón‐Camacho O, Pérez‐González E, et al. Clinical, imaging, and molecular findings in a sample of Mexican families with pantothenate kinase‐associated neurodegeneration. Clin Genet. 2015;87(3):259-265.

18. Mikati MA, Yehya A, Darwish H, Karam P, Comair Y. Deep brain stimulation as a mode of treatment of early onset pantothenate kinase-associated neurodegeneration. Eur J Paediatr Neurol. 2009;13(1):61-64.

19. Ma LY, Wang L, Yang YM, Lu Y, Cheng FB, Wan XH. Novel gene mutations and clinical features in patients with pantothenate kinase‐associated neurodegeneration. Clin Genet. 2015;87(1):93-95.

20. Lyoo CH, Prokisch H, Meitinger T, Lee SY, Kim DH, Lee MS. Anticholinergic‐responsive gait freezing in a patient with pantothenate kinase‐associated neurodegeneration. Mov Disord. 2008;23(2):283-284.

21. Liang TW, Truax AC, Trojanowski JQ, Lee VMY, Stern MB, Kotzbauer PT. Partial deficit of pantothenate kinase 2 catalytic activity in a case of tremor‐predominant neurodegeneration with brain iron accumulation. Mov Disord. 2006;21(5):718-722.

22. Li YF, Li HF, Zhang YB, Wu JM. Novel homozygous PANK2 mutation identified in a consanguineous Chinese pedigree with pantothenate kinase-associated neurodegeneration. Biomed Rep. 2016;5(2):217-220.

23. Lee J-H, Park J, Ryu H-S, et al. Clinical heterogeneity of atypical pantothenate kinase-associated neurodegeneration in Koreans. J Mov Disord. 2016;9(1):20-27.

24. Lee CH, Lu CS, Chuang WL, et al. Phenotypes and genotypes of patients with pantothenate kinase-associated neurodegeneration in Asian and Caucasian populations: 2 cases and literature review. The Scientific World Journal. 2013;2013:7.

25. Kwon K-Y, Lee HM, Kim M, Kang SH, Koh S-B. Long-lasting isolated freezing of gait with good response to methylphenidate: a patient with pantothenate kinase-associated neurodegeneration. Parkinsonism Relat Disord. 2015;21(6):671-672. [26] 26. Kruer MC, Hiken M, Gregory A, et al. Novel histopathologic findings in molecularly-confirmed pantothenate kinase-associated neurodegeneration. Brain. 2011;134(4):947-958.

27. Isaac C, Wright I, Bhattacharyya D, Baxter P, Rowe J. Pallidal stimulation for pantothenate kinase-associated neurodegeneration dystonia. Arch Dis Child. 2008;93(3):239-240.

28. Hartig MB, Hörtnagel K, Garavaglia B, et al. Genotypic and phenotypic spectrum of PANK2 mutations in patients with neurodegeneration with brain iron accumulation. Ann Neurol. 2006;59(2):248-256.

29. Han J, Kim DW, Lee C-H, Han S-H. Optic atrophy in a patient with atypical pantothenate kinase-associated neurodegeneration. J Neuroophthalmol. 2016;36(2):182-186.

30. Hakim A, Rozeik C, Fedorcak M. Pantothenate kinase-associated neurodegeneration (PKAN) in a child with Down syndrome. A case report and follow-up with MRI. BJR case Reports. 2015:20150011.

31. Gothwal S, Nayan S. Hallervorden–spatz syndrome with seizures. Basic clinical Neuroscience Research. 2016;7(2):165-166.

32. Doi H, Koyano S, Miyatake S, et al. Siblings with the adult-onset slowly progressive type of pantothenate kinase-associated neurodegeneration and a novel mutation, Ile346Ser, in PANK2: Clinical features and 99mTc-ECD brain perfusion SPECT findings. J Neurol Sci. 2010;290(1-2):172-176.

33. Diaz N. Late onset atypical pantothenate-kinase-associated neurodegeneration. Case Rep Neurol Med. 2013;2013:860261.

34. Dastsooz H, Nemati H, Fard MAF, Fardaei M, Faghihi MA. Novel mutations in PANK2 and PLA2G6 genes in patients with neurodegenerative disorders: two case reports. BMC Med Genet. 2017;18(1):87.

Chung SJ, Lee JH, Lee MC, Yoo HW, Kim GH. Focal hand dystonia in a patient with PANK2 mutation. Mov Disord. 2008;23(3):466-468.

36. Cheng Y, Liu Y-t, Yang Z-h, Yang J, Shi C-h, Xu Y-m. Novel compound heterozygous PANK2 gene mutations in a Chinese patient with atypical pantothenate kinase-associated neurodegeneration. International Journal of Neuroscience. 2018;128(12):1109-1113.

37. Bozi M, Matarin M, Theocharis I, Potagas C, Stefanis L. A patient with pantothenate kinase-associated neurodegeneration and supranuclear gaze palsy. Clin Neurol Neurosurg. 2009;111(8):688-690.

38. Aryani O, Houshmand M, Fatehi F. A novel PANK2 gene mutation in a Persian boy: the first report from Iran. Clin Neurol Neurosurg. 2013;115(7):1170-1172.

39. Angural A, Singh I, Mahajan A, et al. A variation in PANK2 gene is causing Pantothenate kinase-associated Neurodegeneration in a family from Jammu and Kashmir–India. Scientific reports. 2017;7(1):4834.

40. Akcakaya NH, Iseri SU, Bilir B, et al. Clinical and genetic features of PKAN patients in a tertiary centre in Turkey. Clin Neurol Neurosurg. 2017;154:34-42.

41. Aggarwal A, Schneider SA, Houlden H, et al. Indian‐subcontinent NBIA: unusual phenotypes, novel PANK2 mutations, and undetermined genetic forms. Mov Disord. 2010;25(10):1424-1431.

42. Xue X, Huang X, Wang F, Xu E, Jia J. Clinical and Genetic Diagnosis of Hallervorden－Spatz Disease in 1 Family Zhonghua Yi Chuan Xue Za Zhi. 2013;30(002):255-256.

43. Xu J, Wu X, Lu B, Ma J. A case report of pantothenic kinase-associated neurodegeneration with peripheral nerve damage Lin Chuang Shen Jing Bing Xue Za Zhi. 2016;29(6):479.

44. Li W, Sun Q, Yi F, et al. Clinical characteristics of pantothenate kinase-associated neurodegeneration （report of 1 case）. Lin Chuang Shen Jing Bing Xue Za Zhi. 2018;31(1):61-63.

Zhang L, Wang X, Zou L. Clinical and Genetic Diagnosis of Hallervorden － Spatz Disease in 2 Families. Zhonghua Shi Yong Er Ke Lin Chuang Za Zhi. 2011;26:1276-1278.

46. Zhu J, Z. YC. Clinical feature and genetic analysis of a family of pantothenate kinase associated neurodegeneration [Master thesis]. JiNan，Shan Dong，China, Shandong University; 2011.

47. Shan J, Wen B, Zhu J, Lin P, Zheng J, Yan C. Novel PANK2 gene mutations in two Chinese siblings with atypical pantothenate kinase-associated neurodegeneration. Neurol Sci. 2013;34(4):561-563.

48. Moccia M, Cozzolino A, Cicarelli G, Barone P, Pellecchia MT. Early Cues to Detect Atypical Panthothenate Kinase-Associated Neurodegeneration. The Journal of neuropsychiatry and clinical neurosciences. 2015;27(1):e78-e79.

49. Ghafouri-Fard S, Yassaee VR, Rezayi A, Hashemi-Gorji F, Alipour N, Miryounesi M. A Novel Nonsense Mutation in PANK2 Gene in Two Patients with Pantothenate Kinase-Associated Neurodegeneration. Int J Mol Cell Med. 2016;5(4):255-259.

50. Chan K, Lam C, Lee L, Tong S, Yuen Y. Pantothenate kinase-associated neurodegeneration in two Chinese children: identification of a novel PANK2 gene mutation. Hong Kong Medical Journal. 2008;14(1):70-73.

51. Zhou J, He J, Kou L, et al. Phenotypic and genotypic features of twenty children with classic pantothenate kinase-associated neurodegeneration. Zhonghua Er Ke Za Zhi. 2017;55(9):678-682.

52. Kim S-H, Sung Y-H, Park K-H, et al. Novel compound heterozygous mutations in the pantothenate kinase 2 gene in a Korean patient with atypical pantothenate kinase associated neurodegeneration. J Mov Disord. 2009;2(1):45-47.

53. Nassif D, Pereira JS, Spitz M, Capitão C, Faria A. Neurodegeneration with brain iron accumulation: A case report. Dement Neuropsychol. 2016;10(2):160-164.

54. Song X, Wang Y, Shi Y, et al. Clinical manifestations and detection of pantothenate kinase 2 gene mutation in a patient with Hallervorden-Spatz syndrome. Zhonghua yi xue za zhi. 2009;89(47):3320-3323.

55. Zhang Y, Tang B, Dou R, et al. Clinical, magnetic resonance image features and detection of pantothenate kinase 2 gene mutations in Chinese patients with Hallervorden-Spatz syndrome. Zhonghua Shenjing Ke Za Zhi. 2005;38:34-37.

56. Pan L, Yu L, Yin Y, Xu Y. A novel PANK2 mutation in a 12-year-old Chinese boy with pantothenate kinase-associated neurodegeneration. Neurol India. 2013;61(2):175-176.

57. Houlden H, Lincoln S, Farrer M, Cleland P, Hardy J, Orrell R. Compound heterozygous PANK2 mutations confirm HARP and Hallervorden-Spatz syndromes are allelic. Neurology. 2003;61(10):1423-1426.

58. Zhang Y, Zhou D, Yang T. Novel PANK2 mutation in a Chinese boy with PANK2-associated neurodegeneration: A case report and review of Chinese cases. Medicine. 2019;98(4):e14122.

59. Yapici Z, Akcakaya NH, Tekturk P, Iseri SAU, Ozbek U. A novel gene mutation in PANK2 in a patient with severe jaw-opening dystonia. Brain Dev. 2016;38(8):755-758.

60. Yamashita S, Maeda Y, Ohmori H, et al. Pantothenate kinase-associated neurodegeneration initially presenting as postural tremor alone in a Japanese family with homozygous N245S substitutions in the pantothenate kinase gene. J Neurol Sci. 2004;225(1-2):129-133.

61. Wu YW, Hess CP, Singhal NS, Groden C, Toro C. Idiopathic basal ganglia calcifications: an atypical presentation of PKAN. Pediatr Neurol. 2013;49(5):351-354. 62. He J, Niu ZP. Genetic analysis and clinical study of 15 Chinese children with PANK2-associated neurodegeneration, Shan Xi Medical University; 2013.

63. Marelli C, Piacentini S, Garavaglia B, Girotti F, Albanese A. Clinical and neuropsychological correlates in two brothers with pantothenate kinase–associated neurodegeneration. Mov Disord. 2005;20(2):208-212.

64. Antonini A, Goldwurm S, Benti R, et al. Genetic, clinical, and imaging characterization of one patient with late‐onset, slowly progressive, pantothenate kinase‐associated neurodegeneration. Mov Disord. 2006;21(3):417-418.

65. Assami S, Azzedine H, Nouioua S, et al. Pantothenate kinase–associated neurodegeneration: Clinical description of 10 patients and identification of new mutations. Mov Disord. 2011;26(9):1777-1779.

66. Habibi AH, Razmeh S, Aryani O, et al. A novel homozygous variation in the PANK2 gene in two Persian siblings with atypical pantothenate kinase associated neurodegeneration. Neurology international. 2019;11(1):7959.
